# Depressive symptoms at short‐, medium‐, and long‐term follow‐up after bariatric surgical procedures: A systematic review and meta‐analysis

**DOI:** 10.1111/obr.13927

**Published:** 2025-04-13

**Authors:** Alyssa J. Budin, Wendy A. Brown, Andrew D. MacCormick, Ian Caterson, Priya Sumithran

**Affiliations:** ^1^ Department of Surgery School of Translational Medicine, Monash University, The Alfred Centre Melbourne Victoria Australia; ^2^ Alfred Health The Alfred Centre Melbourne Victoria Australia; ^3^ Department of Surgery The University of Auckland Auckland New Zealand; ^4^ Middlemore Hospital Te Whatu Ora Counties Manukau Otahuhu Auckland New Zealand; ^5^ The Boden Initiative, Charles Perkins Centre The University of Sydney Camperdown New South Wales Australia; ^6^ Department of Endocrinology Royal Prince Alfred Hospital Camperdown New South Wales Australia; ^7^ Department of Endocrinology and Diabetes Alfred Health Melbourne Victoria Australia

**Keywords:** bariatric surgery, depression, patient‐reported measures, psychosocial health

## Abstract

**Importance:**

Patients experience both positive and negative changes in mood following bariatric surgery and mental health outcomes have been reported to differ between procedure types. Understanding changes in symptoms over time and between surgical procedures is vital to providing meaningful, long‐term, patient‐centered care.

**Objective:**

To examine the nature and time course of changes in depressive symptoms after different bariatric procedures.

**Evidence Review:**

Medline, Embase, Emcare, PsycINFO, CINAHL, and CENTRAL databases were systematically searched from inception to January 18, 2024. Ninety publications describing patient‐reported depressive symptoms in 13,146 individuals undergoing bariatric procedures were included.

**Findings:**

Qualitative analysis indicated a reduction of depressive symptoms at all time points following all bariatric procedure types. However, a subset of patients experienced worsening symptoms post‐surgery. Meta‐analyses indicated depressive symptoms improve following bariatric surgery by an SMD of −0.6 (95% CI: −0.8, −0.4) in the short term (0–4 months post‐surgery), −0.9 (95% CI: −1.0, −0.8) in the medium term (5–12 months), and −0.7 (95% CI: −0.9, −0.5) in the long term (> 12 months). There was no evidence that surgery type was associated with the change in depressive symptoms at any time point post‐surgery.

**Conclusions and Relevance:**

Patient‐reported depressive symptoms improve following bariatric surgery with improvements peaking in the medium term and diminishing over time. Significant heterogeneity in the results cannot be explained by surgery type, baseline depression, or depression instrument used across studies. Long‐term management of post‐bariatric surgery patients must consider the potential for adverse psychological effects of surgery.

## 1 INTRODUCTION

Metabolic Bariatric Surgery (MBS) is well‐established as an effective, long‐term treatment for obesity that results in improvements to associated medical conditions as well as overall improvements in quality of life and well‐being. The growing number of patients opting to undergo surgery has facilitated the recognition of mental health disturbances occurring post‐surgery.^1^ A meta‐analysis of 32 studies and several large cohort studies have found the rates of suicide and self‐harm behaviors to be up to four times higher in bariatric cohorts compared to the general population,^2^ with those undergoing Roux‐en‐Y gastric bypass (RYGB) being at higher risk compared to those undergoing adjustable gastric banding (AGB) or vertical banded gastroplasty (VBG).^1^, ^3^, ^4^ Patients undergoing RYGB also demonstrate a threefold higher rate of alcohol and substance use disorders compared to the general population,^5^ with post‐bariatric patients over‐represented in substance abuse treatment programs.^6^, ^7^, ^8^, ^9^ Post‐bariatric patients have also demonstrated worsening or new onset depression^10^, ^11^ and anxiety^12^, ^13^ disorders, with increased hospitalizations for depression observed within 1 year of RYGB.^1^, ^2^, ^14^ There is limited and conflicting evidence regarding the differences between sleeve gastrectomy (SG) and other procedures; however, there appear to be higher rates of adverse mental health outcomes following gastric bypass and sleeve procedures compared to “restrictive” procedures such as AGB and VBG.^3^, ^4^ These population‐level differences in depression, suicide and self‐harm behaviors, and alcohol and substance use between procedure types warrant further investigation to identify any subclinical differences that may be present, as well as to elucidate any potential mechanisms that may underlie these differences.

Bariatric procedures profoundly impact the production and release of gut hormones, the composition of gut microbiota, bile acid circulation, and vagal signaling. There is evidence suggesting that these effects on the gut‐brain axis may not only facilitate changes in appetite and glycemia but could also impact mental functioning including reward processing and mood.^15^ An array of gut peptides that are variably altered after different bariatric procedures have been linked to behaviors indicative of depression and anxiety in pre‐clinical models^16^, ^17^, ^18^, ^19^, ^20^, ^21^, ^22^ with demonstrated effects in brain regions related to stress, reward, and emotional regulation when administered both directly into brain regions and peripherally.^23^, ^24^, ^25^ For instance, ghrelin decreases following SG, and in some studies also RYGB and biliopancreatic diversion procedures (BPD), but not following AGB and VBG procedures.^15^, ^26^ Short‐ and long‐term administration of ghrelin in pre‐clinical models has demonstrated an anti‐depressant effect,^16^, ^17^, ^18^ although there is limited human data.^27^, ^28^ Circulating levels of cholecystokinin (CCK) and glucagon‐peptide 1 (GLP‐1) are increased post‐prandially after SG and RYGB procedures. In addition to their peripheral effects, both act in brain regions involved in stress, reward, and emotional regulation.^29^ CCK has been implicated in anxiety and depression in pre‐clinical models,^30^, ^31^, ^32^ and administration of CCK‐4 is associated with anxiety and panic attacks in humans.^33^, ^34^ Several analyses of real‐world data have examined associations between GLP‐1 receptor agonists and suicidal ideation and behaviors, generally showing a reduction in risk, although results are mixed.^35^, ^36^, ^37^ In addition to these neuroendocrine effects, there is evidence suggesting that gut microbiota composition and circulating bile acids may also influence mood.^38^ MBS has been shown to alter gut microbiota profiles, with distinct differences in composition following RYGB compared to SG, associated with weight loss and adverse events in clinical studies.^39^, ^40^, ^41^, ^42^ Distinct microbiota changes have also been identified in both pre‐clinical research^43^, ^44^, ^45^ and studies of human patients with depression,^46^, ^47^, ^48^, ^49^, ^50^ major depressive disorder (MDD),^51^, ^52^, ^53^ bipolar depression,^54^ post‐partum depression,^55^ and anxiety.^56^, ^57^ In addition, the patients with depression often have altered gastrointestinal functioning similar to that of post‐bariatric patients^58^ with a pathogenic effect of gut dysbiosis being linked to both depression and anxiety.^59^, ^60^, ^61^ Alterations in gut function and bile acids are also linked to changes in drug pharmacokinetics following MBS, whereby temporary or permanent changes in medication absorption are induced by various procedures. The type of procedure plays an important role, with RYGB and other “malabsorptive” procedures being more likely to affect the absorption of drugs^62^, ^63^ including antidepressant and anti‐anxiety medications, which may further contribute to changes in psychosocial well‐being over time.^64^, ^65^, ^66^, ^67^ Taken together, this evidence suggests that altered gut‐brain signaling via several appetite‐regulating hormones and neuropeptides, gut microbiota composition, and circulating bile acids could, directly and indirectly, modulate mood and behavior to contribute to adverse mental health sequelae post‐surgery. Because different bariatric surgeries induce distinct alterations in gut‐brain communication, we hypothesized that depression outcomes would vary between procedure types.

Assessment of depression outcomes in research has traditionally been conducted using medical and prescription records to identify patients with official diagnoses or antidepressant medication use. However, the recent uptake of patient‐reported outcome measures (PROMs) across multiple levels of health care and research has generated a valuable, large‐scale reservoir of data. Collecting depression outcomes via self‐report rating scales is a simple, time‐ and cost‐efficient method to assess large numbers of patients' emotional states over time, and it is often collected as a secondary outcome in large studies investigating bariatric interventions.^68^, ^69^ These data provide valuable insight into the patient's interpretation of their mental health, representing outcomes that are highly valued by patients, with the potential to capture more subtle mood changes that fall below clinical thresholds.^70^ Previous reviews of both clinical and patient‐reported depression outcomes have found an overall improvement in depression following MBS but have not investigated each procedure type separately, nor any such changes over time.^71^, ^72^, ^73^ As such, this review aims to examine how depressive symptoms assessed by PROMs fluctuate after different bariatric procedures at short‐, medium‐, and long‐term follow‐up.

## 2 METHODS

This systematic review and meta‐analysis were conducted according to the Preferred Reporting Items for Systematic Review and Meta‐Analysis (PRISMA)^74^ and Meta‐Analysis of Observational Studies in Epidemiology (MOOSE)^75^ reporting guidelines. The study was prospectively registered on PROSPERO (CRD42021240570).

### 2.1 Search strategy

A systematic literature search was conducted on Medline, Embase, Emcare, PsycINFO, CINAHL, and CENTRAL databases for English‐language articles published from database inception to January 18, 2024. Search terms were related to obesity (e.g., obes* and BMI), bariatric surgery (e.g., bariatric*, sleeve gastrectomy, and gastric bypass), and depression (e.g., depress* and antidepress*) (Table S1). An additional manual search was conducted in the reference lists of identified articles and reviews.

### 2.2 Study selection

All search results were uploaded to Covidence (Veritas Health Innovation, Melbourne, Australia). Studies were included if they were published in English in a peer‐reviewed journal and reported on patients undergoing a surgical or endoscopic bariatric procedure for weight loss, where depressive symptoms were assessed before and after surgery in the same participants using a validated self‐report scale. Exclusion criteria included non‐primary bariatric procedures, patient populations with additional conditions/diseases not commonly associated with obesity (e.g., cancer), additional interventions (e.g., CBT), reviews, case studies, or non‐peer‐reviewed materials. Studies were also excluded if a non‐validated or non‐specific patient‐reported measure was used, depressive symptoms were not reported both before and after surgery, or depressive symptoms were not reported by procedure type.

Authors AB and PS conducted screening of full‐text articles. Discrepancies were reviewed by a third author WB and resolved by discussion and consensus.

### 2.3 Data extraction and study quality

A data extraction sheet was used to extract the following information: study identifiers (author, year of publication, location); study design (aims, design [RCT, cohort, etc.], time period, sample size, bariatric procedure(s), follow‐up, depression assessment tools, data collection methods, and statistics); participant characteristics (age, sex, BMI, and baseline assessments); and outcome data (BMI/weight change, depression before and after surgery, additional outcomes, overall findings, and conclusions). For any depression assessment instrument with scores that increased to indicate improvement in depressive symptoms, aggregated positive scores were made negative. Where data were not presented in text form but only in figures, values were estimated using PlotDigitizer, 3.1.5, 2023 (https://plotdigitizer.com). Risk of bias and quality assessment was assessed using the National Heart, Lung, and Blood Institute (NHLBI) ‘Quality Assessment Tool for Before‐After (Pre‐Post) Studies with no Control Group’ to give an overall rating of poor, fair, or good for each study.

### 2.4 Data synthesis and analysis

Results from multiple articles reporting on the same population of patients were combined into a single cohort for meta‐analysis. The primary outcome was the standardized mean difference (SMD) in depressive symptoms post‐surgery. Several variables were imputed (for example, mean, standard deviation, mean difference, standard error, 95% C.I., *p*‐value) into Review Manager ™ 5.4 (The Cochrane Collaboration) which facilitates the conversion of these values to SMD and 95% C.I. for quantitative synthesis. Methods described by Wan et al were used to estimate the mean and standard deviation in studies reporting the median, interquartile range (IQR), and range for depression outcomes.^76^

As a variety of depression assessment tools were used across studies, quantitative assessment outcomes were reported as SMDs to facilitate comparison between different scales. Effect sizes for SMDs of 0.2, 0.5, and 0.8 were considered small, medium, and large, respectively.^77^ Pooled estimates were obtained using an inverse‐variance weighted random‐effects model.

Post‐operative study data were organized into three follow‐up periods for analysis of depression outcomes over time: Short Term (0–4 months), Medium Term (5–12 months), and Long Term (>12 months). Surgery types were also grouped into (1) Band procedures, (2) Bypass procedures (gastric bypass [unspecified], Roux‐en‐Y Gastric Bypass [RYGB], One‐Anastomosis Gastric Bypass [OAGB]), (3) Sleeve Procedures, (4) Biliopancreatic Diversion with Duodenal Switch (BPD/DS), and (5) Other (Duodeno‐jejunal Bypass Liner [DJBL], Intragastric Balloon [IGB], Vertical‐Banded Gastroplasty [VBG]). For studies that divided results by variables other than surgery type, subgroups were combined to form one group using the sample size and mean (SD) values for meta‐analysis.^78^

Potential sources of heterogeneity were investigated with subgroup and sensitivity analyses. Meta‐regression was used to examine the differences in prevalence estimates between groups. Surgery group, baseline levels of depression, and the depression instrument used were investigated as potential modulators of depressive symptom changes.

Statistical heterogeneity was appreciated using Higgins *I* ^2^ statistic, where values 25%, 50%, and 75% represented low, moderate, and high heterogeneity, respectively.^79^ Publication bias was evaluated by visual inspection of funnel plots and Egger's regression test (*p*‐value < 0.05). Statistical analyses were conducted in Review Manager™ 5.4 and Stata 17 (StataCorp LLC).

### 2.5 Sensitivity analysis

Several sensitivity analyses were performed to assess the strength of the conclusion by adjusting eligibility criteria at each time point. Studies were excluded if they contained only adolescent participants, only female participants, and those rated as poor and/or fair quality. Studies were also excluded if pre‐surgery or post‐surgery mean (SD) values indicated that the depressive symptom score at any timepoint was not normally distributed (if mean minus 3 SDs included a negative value). For each analysis, effect size, statistical significance, and heterogeneity were examined to determine whether the summary estimates differed meaningfully from the main analysis.

## 3 RESULTS

**FIGURE 1:**
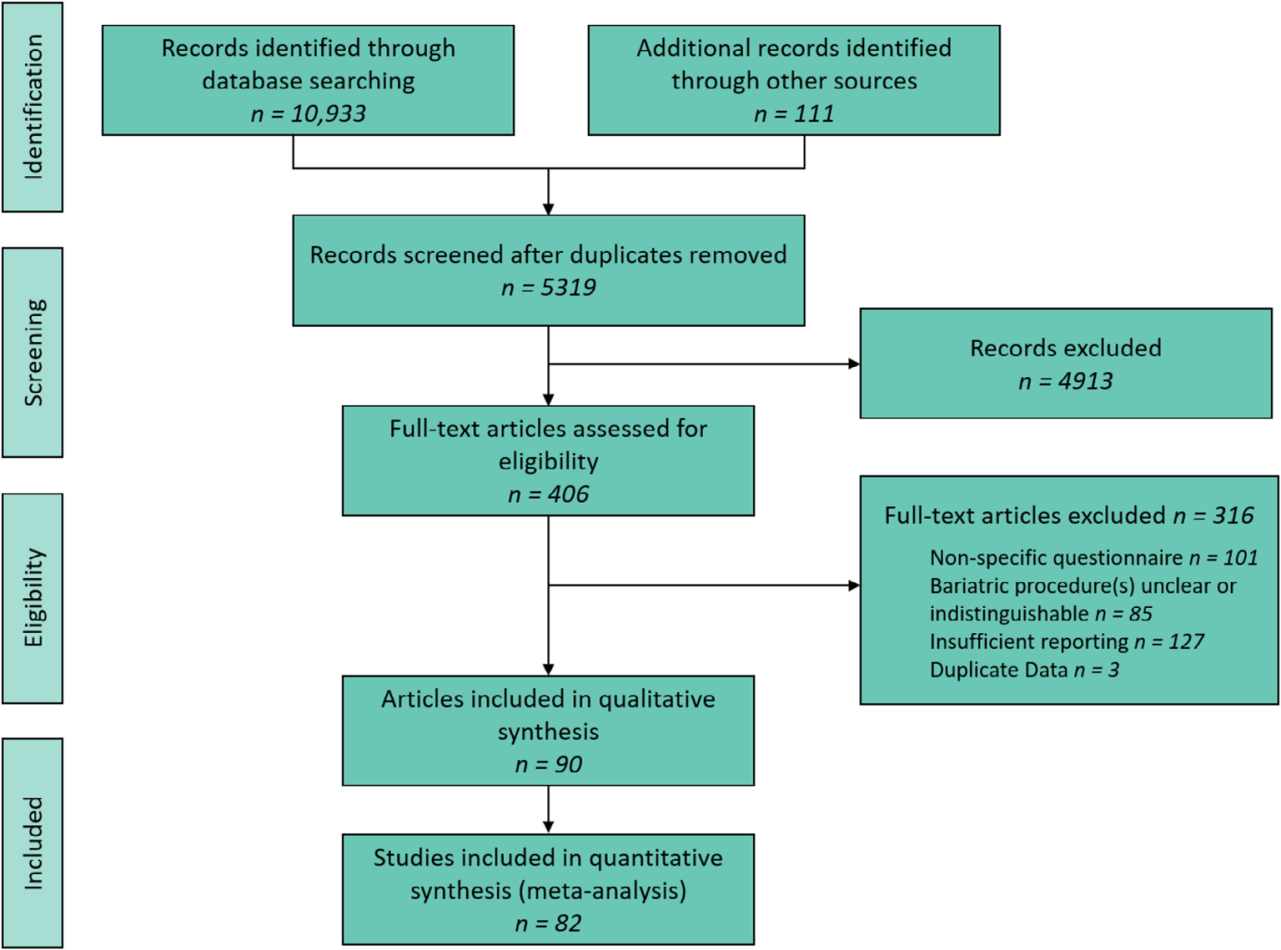
Preferred Reporting Items for Systematic Reviews and Meta‐Analysis (PRISMA) study selection flow diagram.

The initial systematic review yielded 10,933 citations, with manual searches providing an additional 111 articles. Following the removal of duplicates, 5319 articles were screened by title and abstract, identifying 406 relevant articles, of which 90 were included in the qualitative synthesis.^80^, ^81^, ^82^, ^83^, ^84^, ^85^, ^86^, ^87^, ^88^, ^89^, ^90^, ^91^, ^92^, ^93^, ^94^, ^95^, ^96^, ^97^, ^98^, ^99^, ^100^, ^101^, ^102^, ^103^, ^104^, ^105^, ^106^, ^107^, ^108^, ^109^, ^110^, ^111^, ^112^, ^113^, ^114^, ^115^, ^116^, ^117^, ^118^, ^119^, ^120^, ^121^, ^122^, ^123^, ^124^, ^125^, ^126^, ^127^, ^128^, ^129^, ^130^, ^131^, ^132^, ^133^, ^134^, ^135^, ^136^, ^137^, ^138^, ^139^, ^140^, ^141^, ^142^, ^143^, ^144^, ^145^, ^146^, ^147^, ^148^, ^149^, ^150^, ^151^, ^152^, ^153^, ^154^, ^155^, ^156^, ^157^, ^158^, ^159^, ^160^, ^161^, ^162^, ^163^, ^164^, ^165^, ^166^, ^167^, ^168^, ^169^ Fourteen articles were found to report on the same population of participants at different time points and were combined as appropriate to result in a total of 82 studies included in the meta‐analysis (Figure 1).

### 3.1 Study characteristics

An overview of study characteristics is provided in Table 1. Sixty‐three were prospective cohort studies, 18 were retrospective cohort studies, and 1 was a randomized controlled trial. A total of 13,146 surgical participants (range 13–4062) were involved. Four studies were conducted in adolescents, one in both adults and adolescents, 73 only in adults, and four did not identify the age group of participants.

**TABLE 1:** Characteristics and depression outcomes of included studies.

Ref	Author (year)	Setting	No. of women (%)	Age (years)	Baseline BMI	Procedure (n)	Questionnaire	Assessment time points (months)	Depression score	Depression trend^		Retention rate at final follow‐up	Quality rating (of 11)
				Mean (SD)	(kg/m^2^)				Mean (SD)	MD	p		
^26^	Abdelaziz (2023)	Egypt	86 of 108 (79.6)	34.7 (8.6)	40.5 (5.0)	SG (108)	BDI‐II	0	14.7 (10.5)			95.6%	Good (9)
								1	10.6 (7.3)	4.1	<0.001		
^27^	Alabi (2018)	Mexico	56 of 73 (76.7)	38.1 (9.1)	38.8 (3.8)	GB (73)	BDI‐II	0	12.3 (NR)			63.0%	Fair (7)
								6	4.2 (NR)	8.1	0.006		
								12	5 (NR)	7.3	ns		
^28^	Alfonsson (2014)	Sweden	101 of 129 (78.3)	42.8 (10.5)	43.0 (4.0)	RYGB (129)	HADS	0	4.2 (3.1)			100%	Fair (7)
								12	2.7 (3.3)	1.4	<0.001		
^29^	Andersen (2010)	Norway	28 of 50 (56.0)	37.9 (7.9)	51.7 (7.5)	DS/BPDDS (50)	HADS	0	6.3 (4.6)			92.0%	Good (10)
								12	2.1 (2.3)	4.2	<0.001		
^30^	Aasprang (2013)							24	2.1 (3.0)	4.2	<0.001		Good (10)
								60	3.5 (NR)	2.8	<0.001		
^31^	Atwood (2021)	Canada	148 of 179 (82.7)	43.3 (10.4)	49.3 (9.5)	RYGB (179)	PHQ‐9	0	10.3 (6.5)			49.2%	Fair (6)
								12	2.7 (3.6)	7.6	NR		
								24	3.5 (4.0)	6.9	NR		
^32^	Ayloo (2015)	USA	115 of 137 (83.9)	41.6 (10.3)	47.7 (7.7)	AGB (58)	BDI	0	9.9 (7.8)			100%*	Poor (5)
								6	8.6 (9.9)	1.3	SE: 1.0		
						RYGB (28)		0	13.7 (1.1)				
								6	5.6 (5.9)	8.1	SE: 1.1		
						SG (51)		0	11.5 (8.9)				
								6	6.3 (7.3)	5.2	SE: 1.5		
^33^	Barzin (2020)	Iran	581 of 685 (84.8)	38.7 (10.9)	45.1 (6.0)	GB (242)	BDI	0	17.2 (10.5)			100%	Good (10)
								12	11.1 (9.6)	6.1	<0.001		
						SG (443)		0	16.1 (10.2)				
								12	9.6 (8.9)	6.5	<0.001		
^34^	Bawahab (2022)	Saudi Arabia	71 of 105 (67.6)	34.1 (10.3)	45.8 (6.5)	SG (105)	DASS‐21	0	12.7 (10.0)		<0.001^†^	100%	Good (9)
								3	15.1 (12.5)	−2.4			
								6	10.3 (9.6)	2.4			
								12	11.3 (10.0)	1.4			
^35^	Blom‐Høgestøl (2023)	Norway	123 of 197 (75.0)	43.7 (9.7)	43.0 (4.4)	RYGB (197)	HADS	0	3.0 (3.2)			83.0%	Fair (7)
								24	2.0 (2.9)	1.0	<0.001		
^36^	Brancatisano (2008)	Australia	679 of 838 (81.0)	44.0 (8.0)	44.0 (8.0)	AGB (838)	BDI‐II	0	17.3 (10.0)			55.0%	Fair (7)
								12	7.2 (6.0)	10.1	<0.001		
^37^	Buzgova (2016)	Czech Republic	45 of 68 (66.2)	44.2 (9.6)	42.6 (5.4)	GCP (25)	HADS	0	3.3 (3.1)			100%	Good (8)
								3	2.9 (2.7)	0.4	0.231		
								6	3.4 (3.2)	−0.1	0.899		
								12	2.9 (3.9)	0.4	0.329		
						SG (43)		0	5.7 (3.8)				
								3	2.3 (2.6)	3.4	<0.001		
								6	2.4 (2.4)	3.3	<0.001		
								12	3.7 (3.4)	2.0	0.004		
^38^	Calisir (2020)	Turkey	34 of 48 (70.8)	38.9 (11.2)	47.3 (6.3)	SG (48)	HDRS	0	9.1 (5.8)			100%	Fair (7)
								6	4.4 (3.7)	4.7	<0.001		
								12	2.2 (1.9)	6.9	<0.001		
^39^	Canetti (2016)	Israel	44 of 51 (86.3)	45.6 (9.8)	46.1 (8.9)	SRVG (51)	MHI	0	52.1 (16.9)			70.6%	Fair (8)
								12	49.7 (18.0)	2.5	ns		
								120	59.5 (21.5)	−7.4	0.004		
^40^	Castellini (2014)	Italy	75 of 83 (90.4)	45.3 (10.1)	NR	AGB (27)	BDI	0	16.0 (11.1)			100%*	Good (10)
								12	6.7 (6.5)	9.3	<0.01		
						BPD (26)		0	17.8 (12.1)				
								12	9.9 (6.6)	7.9	<0.05		
						RYGB (30)		0	18.3 (11.3)				
								12	8.5 (6.5)	9.8	<0.01		
^41^	Chahal‐Kummen (2021)	Norway	181 of 249 (72.7)	41.2 (11.0)	43.9 (6.0)	SG (249)	HADS	0	4.6 (6.3)	2.1	<0.001	83.1%	Good (8)
								24	2.6 (3.4)				
^42^	Chahal‐Kummen (2023)		169 of 236 (71.6)	44.0 (9.2)	43.2 (5.9)	RYGB (236)		0	3.1 (3.2)	0.8	ns	88.6%	Good (8)
								24	2.3 (3.0)				
^43^	Colles (2008)	Australia	103 of 129 (79.8)	45.2 (11.5)	44.3 (6.8)	AGB (129)	BDI	0	15.4 (8.3)			75.0%	Fair (8)
								12	7.4 (5.3)	8.0	<0.001		
^44^	Deliopoulou (2013)	Greece	100 of 100 (100)	37.5 (11.8)	43.5 (9.5)	IGB (100)	BDI‐II	0	20.3 (8.5)			100%	Poor (6)
								3	10.7 (7.2)	9.6	<0.001		
								6	7.9 (5.6)	12.4	<0.001		
^45^	deMeireles (2020)	USA	3272 of 4062 (80.6)	46.6 (11.7)	47.2 (8.2)	AGB (9)	BODY‐Q~	0	76.7 (28.4)			62.5%	Good (8)
								12	84.1 (22.6)	7.4	0.257		
						BPD/DS (19)		0	62.7 (17.2)				
								12	80.0 (12.6)	17.3	0.0008		
						RYGB (637)		0	70.5 (21.1)				
								12	78.8 (21.9)	8.2	<0.0001		
						SG (3386)		0	70.9 (20.8)				
								12	78.0 (22.1)	7.1	<0.0001		
^46^	Dixon (2002)	Australia	33 of 50 (66.0)	NR	48.2 (8.0)	AGB (50)	BDI	0	16.3 (6.5)			100%*	Poor (5)
								12	9.6 (9.0)	6.7	0.009		
^47^	Dixon (2003)	Australia	217 of 262 (82.8)	40.6 (10.0)	44.4 (7.0)	AGB (262)	BDI	0	17.2 (9.0)			100%*	Fair (9)
								12	7.8 (7.0)	9.4	<0.001		
^48^	Dixon (2016)	USA	135 of 149 (90.6)	Md: 40 (NR)	35.6 (2.5)	AGB (149)	BDI‐II	0	8.8 (6.9)			59.8%	Good (9)
								12	3.2 (5.7)	5.6	<0.001		
								24	3.0 (4.7)	5.8	<0.001		
								36	3.1 (6.0)	5.7	<0.001		
								48	3.1 (5.5)	5.7	<0.001		
								60	3.1 (6.7)	5.7	<0.001		
^49^	Dymek (2001)	USA	26 of 32 (81.3)	39.1 (8.47)	56.7 (11.5)	RYGB (32)	BDI	0	14.9 (6.9)			62.5%	Fair (8)
								0.5	8.2 (5.2)	6.7	<0.05		
								6	2.7 (3.8)	12.2	<0.05		
^50^	Emery (2007)	USA	13 of 13 (100)	46.9 (5.7)	51.3 (6.3)	RYGB (13)	BDI	0	15.8 (1.3)			100%	Poor (6)
								12	6.8 (4.6)	9.0	<0.05		
^51^	Erden (2015)	Turkey	31 of 31 (100)	41.4 (8.3)	49.7 (7.9)	SG (31)	BDI	0	11.6 (8.0)			100%*	Fair (7)
								6	2.9 (4.7)	8.6	<0.001		
^52^	Erden (2016)	Turkey	33 of 51 (64.7)	36.9 (9.3)	47.7 (7.6)	SG (51)	BDS	0	10.9 (7.5)			89.5%	Good (10)
								6	2.6 (4.2)	8.4	<0.001		
^53^	Ferreira Pinto (2017)	Brazil	51 of 60 (85.0)	34.7 (9.2)	46.0 (7.5)	RYGB (60)	BDI‐SF	0	9.8 (7.0)			100%	Fair (8)
								16	4.7 (4.6)	5.1	0.001		
^54^	Fischer (2007)	USA	116 of 144 (80.6)	40.3 (NR)	54.2 (NR)	RYGB (144)	BDI	0	16.5 (10.4)#			100%*	Poor (5)
								8	5.9 (6.6)#	10.6	NR		
^55^	Gezer (2023)	Turkey	16 of 22 (72.7)	31.2 (7.8)	46.8 (7.8)	SG (22)	BDI	0	16.2 (7.1)			71.0%	Fair (8)
								12	11.9 (8.0)	4.3	0.066		
^56^	Green (2004)	USA	48 of 65 (73.8)	39.3 (9.9)	54.8 (10.1)	RYGB (65)	BDI	0	16.0 (9.9)			100%	Fair (8)
								6	5.7 (5.3)	10.3	<0.001		
^57^	Grilo (2006)	USA	122 of 137 (89.1)	42.3 (10.2)	51.8 (7.9)#	GB (137)	BDI	0	13.9 (8.3)#			100%	Poor (4)
								12	5.7 (6.3)#	8.2	<0.001		
^58^	Guedes (2016)	Brazil	NR	34.6 (7.1)	40.1 (6.3)	IGB (50)	BDI	0	15.7 (8.2)#			78.0%	Fair (7)
								2	8.8 (7.5)#	6.9	NR		
								4	7.9 (7.2)#	7.9	NR		
								6	9.3 (9.4)#	6.5	0.002		
							HADS‐D	0	7.1 (3.0)#				
								2	5.3 (3.9)#	1.8	NR		
								4	5.7 (3.9)#	1.3	NR		
								6	5.2 (5.0)#	1.8	0.035		
^59^	Hafner (1990)	Australia	118 of 118 (100)	NR	42.7 (6.0)	GB (118)	CCEI	0	5.4 (3.1)			77.1%	Fair (6)
								48	5.0 (3.0)	0.4	ns		
^60^	Hancock (2018)	UK	NR	45.9 (7.2)	NR	AGB (31)	HADS	0	8.5 (4.3)#		0.001^†^	58.1%	Fair (7)
								6	5.7 (4.8)#	2.8			
								12	5.0 (4.9)#	3.5			
								24	4.8 (4.5)#	3.6			
								36	5.1 (4.5)#	3.4			
								48	5.2 (5.0)#	3.2			
								60	6.2 (5.4)#	2.2			
^61^	Hayden (2011)	Australia	219 of 258 (84.9)	41.4 (9.3)	43.8 (8.0)#	AGB (258)	BDI	0	20.1 (10.2)#			100%*	Fair (7)
								12	6.7 (8.1)#	13.4	<0.05^‡^		
^62^	Hosseini (2023)	Iran	53 of 86 (67.1)	35.2 (12.0)	39.3 (6.3)	SG (86)	DASS‐21	0	7.5 (4.6)			91.9%	Fair (8)
								3	4.6 (3.2)	3.0	<0.001		
								6	3.1 (2.9)	4.5	<0.001		
^63^	Ivezaj (2015)	USA	94 of 107 (87.9)	42.7 (10.5)	51.7 (7.8)	GB (107)	BDI	0	13.1 (7.8)			100%	Fair (4)
								6	5.4 (5.3)	7.7	NR		
								12	5.0 (5.5)	8.1	NR		
^64^	Järvholm (2011)	Sweden	25 of 37 (67.6)	16.6 (1.3)	46.5 (5.9)	RYGB (37)	BYI	0	15.8 (10.3)			72.0%	Good (8)
								4	12.3 (9.5)	3.5	0.02		
^65^	Järvholm (2015)		57 of 88 (65.0)	16.8 (1.2)	45.6 (NR)	RYGB (88)		0	14.1 (8.3)	1.7			Good (8)
								12	9.5 (NR)	6.3	<0.001		
								24	9.9 (NR)	5.9	0.001		
^66^	Klemencic (2021)	Slovenia	12 of 19 (63.2)	17.2 (1.2)	NR	DJBL (19)	YSR	0	3.7 (2.9)			63.2%	Fair (7)
								12	2.8 (2.6)	1.0	0.043		
								24	2.9 (2.6)	0.8	0.745		
^67^	Kruseman (2010)	Switzerland	131 of 141 (92.9)	40.0 (10.0)	46.0 (7.0)	RYGB (141)	HADS‐D	0	6.4 (3.7)			56.7%	Good (8)
								96	5.5 (4.4)	0.9	ns		
^68^	Leombruni (2007)	Italy	32 of 38 (84.2)	39.8 (9.9)	43.5 (5.5)	VBG (38)	BDI	0	9.0 (4.2)			100%	Good (8)
								6	4.1 (3.3)	4.9	<0.001		
^69^	Lier (2013)	Norway	94 of 127 (74.0)	41.3 (10.3)	45.3 (5.2)	RYGB (127)	BDI	0	10.9 (9.6)			68.5%	Good (7)
								12	5.9 (9.0)	5.0	0.001		
^71^	Malone (2004)	USA	91 of 109 (83.5)	45.2 (10.1)	47.7 (17.3)#	GB (109)	BDI	0	11.6 (8.8)#			51.4%	Fair (6)
								12	5.4 (6.7)#	6.3	< 0.05^‡^		
^72^	Mamplekou (2005)	Greece	45 of 59 (76.3)	37.7 (10.7)	47.7 (8.0)	VBG (59)	SCL‐90	0	1.5 (0.8)			98.3%	Fair (8)
								24	1.1 (0.5)	0.4	<0.001		
^73^	Masheb (2006)	USA	129 of 145 (89.0)	42.1 (10.3)	51.6 (7.5)	GB (145)	BDI	0	13.5 (7.8)			100%	Fair (5)
								6	5.9 (5.5)	7.6	<0.001		
^74^	Masheb (2007)	USA	122 of 137 (89.1)	42.3 (10.2)	51.8 (7.9)	GB (137)	BDI	0	13.9 (8.2)			100%	Good (7)
								12	5.7 (6.3)	8.2	<0.001		
^75^	Mathus‐Vliegen (2004)	The Netherlands	34 of 50 (68.0)	35.0 (7.4)	50.7 (8.5)	AGB (25)	CES‐D	0	19.4 (11.0)			98.0%	Good (9)
								12	14.6 (13.1)	4.8	<0.01		
^76^	Matini (2014)	Iran	63 of 67 (94.0)	36.8 (8.5)	48.8 (4.7)	GB (67)	HAM‐D	0	5.9 (0.7)			95.5%	Fair (7)
								6	6.3 (0.8)	−0.4	0.311		
^77^	Musselman (2019)	USA	19 of 19 (100)	37.0 (9.0)	49.5 (SE: 0.9)	RYGB (19)	ZDRS	0	54.8 (10.5)			100%*	Fair (8)
								6	45.1 (10.1)	9.7	<0.05		
^78^	Nandrino (2020)	France	106 of 136 (77.9)	41.8 (10.1)	47.9 (6.3)	AGB (136)	BDI	0	15.4 (0.8)			100%*	Fair (9)
								60	9.2 (0.8)	6.2	<0.001		
^79^	Nickel (2005)	Germany & Austria	22 of 22 (100)	38.0 (9.5)	47.4 (7.8)	AGB (22)	HADS	0	8.6 (4.5)		<0.01^†^	CD	Poor (4)
								36	5.3 (4.1)	3.3			
^80^	Nickel (2007)		21 of 21 (100)					48	5.2 (1.1)	3.4			Poor (4)
								60	5.0 (0.8)	3.6			
								72	4.8 (0.7)	3.8			
^81^	Ortega (2012)	Spain	46 of 60 (76.7)	44.1 (10.9)	44.9 (6.3)	RYGB (60)	SCL‐90	0	1.3 (1.1)			100%*	Fair (7)
								6	1.0 (1.0)	0.2	ns		
								12	1.0 (0.9)	0.2	ns		
^82^	Papageorgiou (2002)	Greece	39 of 53 (73.6)	NR	53.8 (9.5)	VBG (53)	SCL‐90	0	2.0 (1.0)			66.1%	Fair (5)
								12	1.6 (0.9)	0.4	0.123		
^83^	Pasi (2023)	Switzerland	35 of 46 (76.1)	44.1 (12.5)	42.4 (5.7)	SG (26)	BDI	0	8.0 (NR)			82.1%	Good (8)
								1	7.1 (4.1)	5.8	ns		
								6	6.7 (7.7)	6.2	ns		
						RYGB (20)		0	12.9 (8.0)				
								1	12.2 (10.7)	0.7	ns		
								6	11.2 (9.8)	1.7	ns		
^84^	Pinto (2017)	Brazil	51 of 60 (85.0)	34.7 (9.2)	46.0 (7.5)	RYGB (60)	BDI	0	9.8 (7.0)			100%	Good (9)
								16	4.7 (4.6)	5.1	0.001		
^85^	Preiss (2018)	Australia	79 of 99 (79.8)	42.6 (9.9)	42.6 (7.6)	AGB (99)	BDI‐II	0	15.7 (10.0)			89.0%	Fair (9)
								1	6.9 (SE: 0.9)	8.8	<0.05^‡^		
								2	6.5 (SE: 0.9)	9.2	<0.05^‡^		
								3	5.9 (SE: 0.9)	9.8	<0.05^‡^		
								4	5.4 (SE: 0.9)	10.3	<0.05^‡^		
								5	5.5 (SE: 0.8)	10.2	<0.05^‡^		
								6	5.5 (8.9)	10.2	<0.05^‡^		
^86^	Pyykko (2021)	The Netherlands	98 of 126 (77.8)	46.4 (10.8)	39.1 (3.5)	GB (126)	CES‐D	0	8.2 (6.4)			100%*	Fair (8)
								12	6.6 (7.5)	1.6	0.007		
^87^	Ribeiro (2022)	Portugal	42 of 42 (100)	41.0 (11.9)	47.7 (5.9)	SG (21)	SCL‐90‐R	0	17.6 (10.0)			100%*	Poor (6)
								6	8.6 (5.6)	9.0	<0.001		
^89^	Rosenberger (2011)	USA	116 of 131 (88.5)	42.9 (10.3)	51.6 (8.0)	GB (131)	BDI	0	13.8 (8.4)			100%*	Fair (6)
								12	4.9 (5.5)	8.9	<0.001		
^90^	Ryden (1996)	Sweden	16 of 20 (80.0)	42 (9.9)	42.0 (9.9)	VBG (20)	BDI	0	10.2 (27.8)			100%*	Fair (8)
								36	4.6 (20.9)	5.6	0.07		
							HSCL	0	0.9 (1.4)				
								36	0.6 (1.2)	0.3	ns		
^91^	Sarwer (2015)	USA	0 of 32 (0)	Md: 48	Md: 45.1 IQR: 42.0, 52.2	RYGB (32)	BDI	0	6.5 (5.9)			96.9%	Fair (8)
								12	4.1 (5.7)	2.4	0.103		
								24	4.0 (7.1)	2.5	0.203		
								36	2.1 (3.6)	4.4	<0.001		
								48	4.1 (5.1)	2.4	0.085		
^92^	Schowalter (2008)	Germany	201 of 248 (81.0)	38.5 (10.1)	46.4 (6.8)	AGB (128)	BDI	0	15.8 (11.3)			31.3%	Fair (8)
								67	9.1 (11.0)	6.7	<0.001		
^93^	Sellberg (2018)	Sweden	69 of 69 (100)	39.5 (NR)	39.2 (3.2)	RYGB (69)	HADS‐D	0	5.1 (3.2)			50.7%	Fair (5)
								9	1.7 (1.8)	3.4	<0.001		
								48	3.5 (3.5)	1.6	0.03		
^94^	Strain (2014)	USA	76 of 105 (72.4)	43.5 (10.8)	50.7 (9.6)	AGB (18)	BDI	0	8.3 (6.9)			100%*	Poor (5)
								22	3.7 (2.8)	4.6	0.04		
						BPD/DS (18)		0	11.7 (9.3)				
								26	3.3 (4.4)	8.4	0.0001		
						RYGB (46)		0	11.1 (8.6)				
								19	4.5 (4.0)	6.6	0.0001		
						SG (23)		0	10.5 (8.9)				
								36	5.5 (5.9)	4.6	0.01		
^95^	Strain (2017)	USA	192 of 275 (69.8)	42.7 (10.0)	53.4 (11.4)	BPD/DS (275)	BDI	0	13.9 (9.5)			24.7%	Poor (7)
								12	7.2 (11.1)	6.7	NR		
								36	7.2 (8.7)	6.7	NR		
								60	7.9 (10.9)	6.0	NR		
								84	8.6 (12.07)	5.3	NR		
								108	5.7 (6.4)	8.2	NR		
^96^	Svanevik (2023)	Norway	32 of 55 (58.0)	47.1 (10.2)	42.1 (5.3)	SG (55)	BDI	0	15.2 (10.8)			85.3%	Fair (8)
								36	9.5 (8.2)	5.7	NR		
			40 of 54 (74.0)	48.2 (8.9)	42.4 (5.4)	GB (54)		0	13.3 (8.6)				
								36	7.7 (7.9)	5.6	NR		
^97^	Sysko (2012)	USA	73 of 101 (72.3)	15.8 (1.1)	47.2 (SE: 0.9)	AGB (101)	BDI	0	7.5 (1.1)		<0.05^†^	100%	Fair (8)
								1	6.7 (SE: 1.2)	0.8			
								3	5.4 (SE: 0.9)	2.2			
								6	4.2 (SE: 1.2)	3.4			
								9	4.0 (SE: 1.0)	3.6			
								12	4.7 (SE: 1.1)	2.8			
^98^	Tan (2021)	Singapore	35 of 55 (63.6)	44.7 (9.4)	40.9 (6.0)	RYGB (20)	HADS	0	3.2 (2.7)			78.6%	Fair (7)
								6	2.4 (2.5)	0.8	NR		
								12	2.6 (3.2)	0.7	NR		
						SG (35)		0	4.0 (3.3)				
								6	2.5 (3.1)	1.4	NR		
								12	2.6 (2.9)	1.4	NR		
^99^	Teufel (2012)	Germany	33 of 70 (64.7)	43.8 (9.1)	51.3 (8.7)	SG (70)	PHQ‐9	0	9.0 (6.8)			100%*	Fair (9)
^88^	Rieber (2013)		28 of 40 (70.0)	43.2 (8.9)	50.9 (8.2)	SG (40)		12	4.2 (4.4)	4.8	<0.001		Fair (5)
^70^	Mack (2016)		48 of 75 (64.0)	45.2 (11.6)	48.7 (8.4)	SG (75)		48	6.0 (5.9)	3.0	0.002		Poor (5)
^100^	Thonney (2010)	Switzerland	43 of 43 (100)	39.2 (1.4)	44.7 (0.4)	GB (43)	BDI‐II	0	13.7 (1.3)			100%	Poor (5)
								12	9.7 (2.0)	4.0	<0.01		
								24	9.3 (1.7)	4.4	<0.01		
							HADS	0	7.0 (0.5)				
								12	4.1 (0.9)	2.9	<0.01		
								24	3.2 (0.6)	3.8	<0.01		
^101^	Tuli (2024)	USA	16 of 21 (76.2)	18.4 (0.4)	Md: 47.5IQR: 42.1, 52.4	SG (21)	BDI‐II	0	8.0 (8.9)			100%	Fair (7)
								12	4.4 (8.4)	3.4	ns		
								24	8.4 (12.7)	−0.8	ns		
^102^	Uruc (2016)	Turkey	53 of 53 (100)	34.8 (9.4)	47.4 (6.4)	SG (53)	BDI	0	14.7 (4.7)			100%	Fair (7)
								6	9.2 (2.8)	5.5	0.001		
^103^	Van Hout (2008)	The Netherlands	91 of 104 (87.5)	38.4 (8.3)	45.4 (5.1)	VBG (104)	SCL‐90	0	24.8 (8.6)			79.4%	Fair (7)
								6	20.8 (6.3)	4.0	<0.001		
								12	20.9 (7.1)	3.9	<0.05^‡^		
								24	23.1 (11.3)	1.7	ns		
^104^	Velcu (2005)	USA	36 of 41 (87.8)	32.4 (3.6)	53.4 (8.9)	RYGB (41)	BDI‐II	0	16.3 (4.1)			100%*	Fair (8)
								60	10.5 (3.2)	5.8	<0.01		
^105^	Vetrovsky (2021)	Czech Republic	20 of 26 (76.9)	45.4 (9.0)	45.1 (7.4)	GB (26)	HADS	0	6.2 (3.9)			100%	Fair (8)
								1	3.7 (2.7)	2.5	SE: 0.56		
								3	3.1 (3.0)	3.1	SE: 0.61		
								6	3.2 (3.2)	3.0	SE: 0.64		
^106^	Vreeken (2023)	The Netherlands	124 of 146 (84.9)	46.1 (5.7)	Md: 41.2IQR: 36.7, 44.0	RYGB (146)	BDI	0	9.0 (6.0)			95.4%	Good (9)
								6	5.0 (3.0)	4.0	<0.001		
^107^	Wang (2022)	China	18 of 30 (60.0)	28.0 (1.3)	38.1 (0.8)	SG (30)	HAM‐D	0	9.5 (1.2)			76.7%	Good (9)
								12	5.1 (0.8)	4.4	<0.001		
^108^	Waters (1991)	USA	132 of 157 (84.1)	36.3 (9.0)	NR	GB (157)	HIS‐GWB	0	7.8 (3.5)			11.5%	Fair (6)
								6	5.7 (2.9)	2.1	<0.001		
								12	6.2 (3.1)	1.6	<0.001		
								24	6.8 (3.5)	0.9	ns		
								36	6.1 (2.9)	1.7	ns		
^109^	White (2006)	USA	124 of 139 (89.2)	42.4 (10.2)	51.7 (7.9)	GB (139)	BDI	0	13.9 (8.2)#			100%	Poor (5)
								12	5.7 (6.2)#	8.2	<0.001		
^110^	White (2010)	USA	311 of 361 (86.1)	43.7 (10.0)	51.1 (8.3)	GB (361)	BDI	0	14.9 (9.5)			47.4%	Poor (4)
								12	8.7 (15.0)	6.2	NR		
								24	8.5 (16.8)	6.4	NR		
^111^	Wimmelmann (2016)	Denmark	28 of 40 (70.0)	39.5 (8.9)	42.7 (SE: 0.7)	RYGB (40)	SCL‐90	0	1.8 (0.6)			67.5%	Fair (8)
								4	1.5 (0.6)	0.3	<0.05		
								18	1.3 (0.5)	0.5	<0.05		
^112^	Winzer (2020)	Austria	32 of 39 (82.1)	42.5 (12.5)	44.2 (4.1)	OAGB (39)	BDI	0	21.1 (9.6)			84.6%	Good (8)
								6	14.0 (7.7)	7.1	0.001		
								12	16.0 (11.3)	8.1	0.011		
^113^	Zeller (2009)	USA	20 of 31 (64.5)	16.4 (1.4)	63.5 (10.6)	RYGB (31)	BDI	0	15.2 (12.4)		<0.05^†‡^	87.5%	Good (8)
								6	7.3 (9.0)	7.9			
								12	5.3 (5.6)	9.9			
^114^	Zeller (2011)	USA	10 of 16 (62.5)	16.2 (1.4)		RYGB (16)		18	9.3 (8.0)	5.9			Good (8)
								24	7.7 (9.7)	7.5			
^115^	Zeller (2017)	USA	9 of 14 (64.3)	16.0 (1.3)	59.2 (8.9)	RYGB (14)	YSR/ASR	0	64.1 (13.0)			82.4%	Good (8)
								6	58.0 (10.2)	6.1	NR		
								12	55.1 (NR)	9.0	NR		
								18	58.1 (8.0)	6.0	NR		
								24	56.6 (9.8)	7.5	NR		
								72	55.6 (7.0)	8.5	NR		

*Note*: Total number and number of surgical patients may differ because of the presence of non‐surgical control groups in certain studies.

NR = No precision measure reported, Md = median, IQR = interquartile range, ns = not significant (no *p*‐value reported).

# Mean and SD of 2 or more groups combined.

* Only those patients with data at all time points were included in the analysis.

^ Depression trend (mean difference and *p*‐value) for each time point compared to baseline.

~ Scale is reverse scored: an increase in scores = an improvement in depressive symptoms.

^†^ *P*‐value represents change over time (no *p*‐values reported for individual time points).

^‡^ Significant (specific *p*‐value not reported).

The mean age was 41 years (± 12.8) and the mean preoperative BMI ranged from 35.6 to 63.5 kg/m^2^. Studies consisted of a majority of female patients (mean 77.3% [± 0.2], range 0–100%). The patients underwent RYGB (28 studies, 2816 patients), SG (21 studies, 4962 patients), AGB (17 studies, 2485 patients), VBG (six studies, 325 patients), BPD/DS (five studies, 388 patients), IGB (two studies, 150 patients), OAGB (1 study, 39 patients), and Greater Curve Plication (GCP; 1 study, 25 patients), and 16 studies reported on 2046 patients undergoing “Gastric Bypass” without defining a specific procedure type.

### 3.2 Assessment of depression

Depression outcomes are summarised in Table 1. Follow‐up time points ranged from 1 to 120 months post‐surgery, with the most collected time point being 12 months (49 studies). The most common measures of depressive symptoms were the Beck Depression Inventory (BDI) used in 50 studies, the depression subscale of the Hospital Anxiety and Depression Scale (HADS‐D) used in 13 studies, and the Symptom Checklist‐90 (SCL‐90) used in six studies. The remaining studies used a mix of tools including the BODY‐Q, Crown‐Crisp Experiential Index (CCEI), Center for Epidemiologic Studies Depression Scale (CED‐D), Depression Anxiety Stress Scale (DASS‐21), Hamilton Depression Inventory (HAM‐D), Health Insurance Study – General Well‐Being Scale (HIS‐GWB), Hopkins Symptom Checklist (HSCL), Mood Adjective Checklist (MACL), Mental Health Inventory (MHI), Patient‐Health Questionnaire (PHQ‐9), Youth/Adult Self‐Report (YSR/ASR), and Zung Depression Rating Scale (ZDRS).

### 3.3 Risk of bias

Following assessment using the NHLBI tool, 27 articles were rated as good, 48 as fair, and 15 as poor (Tables 1 and S2). Many papers did not define the study population, specific inclusion and exclusion criteria, the specific procedure performed, or participant ethnicity adequately. The overall retention rate ranged from 11.5% to 100%, with 27 articles reporting loss to follow‐up of more than 20% of participants (Table 1). One paper did not report the number of participants lost or excluded from follow‐up, 11 articles did not adjust for those lost to follow‐up in their analyses, and 25 reported only on participants for whom they had complete data sets.

### 3.4 Changes in depressive symptoms following metabolic bariatric surgery

A summary of findings is presented in Table 1. The majority of studies reported an overall reduction in depressive symptoms following all types of bariatric procedure compared to pre‐surgery. Changes were observed as early as 2 weeks post‐surgery^103^ and were maintained in several studies out to 5–8 years after surgery.^83^, ^84^, ^102^, ^132^, ^133^, ^144^, ^147^, ^158^, ^167^, ^168^, ^169^

Despite overall improvements in depressive symptoms, several studies identified that post‐surgical depressive symptoms remained elevated compared to population norms, with studies reporting clinically important levels of depression in patients 2 years post‐surgery.^113^, ^118^, ^119^ This was not evident in adolescent cohorts who remained within normal age‐ and gender‐specific norms^167^, ^168^, ^169^ or displayed no significant change in depressive symptoms over 2 years post‐surgery compared to a non‐surgical control group.^155^ In addition, several studies identified a subset of patients experiencing worsening or new symptoms of depression and associated mental health concerns such as self‐harm behaviors and suicidal thoughts following “Gastric Bypass,” RYGB, and SG procedures.^80^, ^101^, ^117^, ^118^, ^119^, ^151^, ^152^, ^153^ One study measured post‐operative depression outcomes only in those who demonstrated baseline scores that were indicative of depression, preventing any new development of depression symptomology from being detected.^98^

Included studies identified associations between depressive symptoms and a variety of other factors, including weight loss,^80^, ^88^, ^97^, ^100^, ^101^, ^103^, ^112^, ^117^, ^121^, ^129^, ^138^, ^142^, ^144^, ^149^ eating‐related behaviors^97^ such as night eating,^107^ emotional eating,^108^, ^131^ overeating,^120^ grazing,^151^ loss of control eating,^151^ and binge eating,^110^, ^124^, ^163^ as well as ADHD,^82^ unemployment,^86^ chronic abdominal pain,^95^ older age,^88^, ^99^, ^101^ reduced cognitive function,^160^ living alone,^144^ female sex,^99^, ^101^ cardiovascular risk factors,^104^ cardiopulmonary fitness,^159^ female sexual dysfunction,^105^ sexual abuse,^111^ anger,^118^, ^119^ persistent excessive daytime sleepiness,^137^ reduced self‐esteem,^134^, ^138^ body dissatisfaction,^138^ reduced physical activity,^141^ higher serum neuregulin‐1 levels,^80^ and the presence of any surgical complications.^99^

#### 3.4.1 Depressive symptoms over time

Fourteen studies reported on the short‐term post‐surgery phase (0–4 months post‐operatively), of which 12 found a reduction in depressive symptoms compared to baseline,^80^, ^91^, ^98^, ^103^, ^112^, ^116^, ^118^, ^119^, ^138^, ^149^, ^159^, ^165^ although four did not provide any measures to determine statistical significance.^112^, ^116^, ^149^, ^159^ One study reported no significant change in depressive symptoms in the first month post‐operatively,^136^ and one reported an increase in depressive symptoms at 3 months.^88^

At medium‐term follow‐up (5–12 months), 57 of 68 (84%) studies reported a reduction in depressive symptoms, although 17 did not provide a measure of significance^81^, ^85^, ^86^, ^88^, ^93^, ^108^, ^114^, ^115^, ^117^, ^124^, ^147^, ^149^, ^150^, ^157^, ^159^, ^164^, ^167^, ^168^, ^169^ and six reported non‐significant decreases.^93^, ^109^, ^129^, ^134^, ^135^, ^143^ At 12 months post‐surgery, several studies observed a subset of patients who had no change or an increase in their depressive symptoms,^117^, ^150^ or that the improvement in depressive symptoms had begun to decelerate.^149^

Thirty‐five articles reported on long‐term depression outcomes (>12 months) with mixed results. Twenty‐six studies (74%) reported a decrease in depressive symptoms with eight not providing a measure of significance^85^, ^114^, ^132^, ^147^, ^148^, ^157^, ^164^, ^167^, ^168^, ^169^ and seven reporting non‐significant differences.^113^, ^114^, ^120^, ^121^, ^142^, ^143^, ^162^ Several studies with multiple follow‐up points reported the initial reduction in depressive symptoms observed from 0 to 12 months were not maintained over time,^83^, ^84^, ^155^, ^157^, ^162^, ^168^, ^169^ with one study observing a significant increase in depressive symptoms at long‐term follow‐up (120 months) compared to baseline following an initial improvement at 12 months^93^ and another observing an increase in the number of adolescent participants within clinical depression at 2 years.^155^

#### 3.4.2 Meta‐analysis of depression outcomes

**FIGURE 2:**
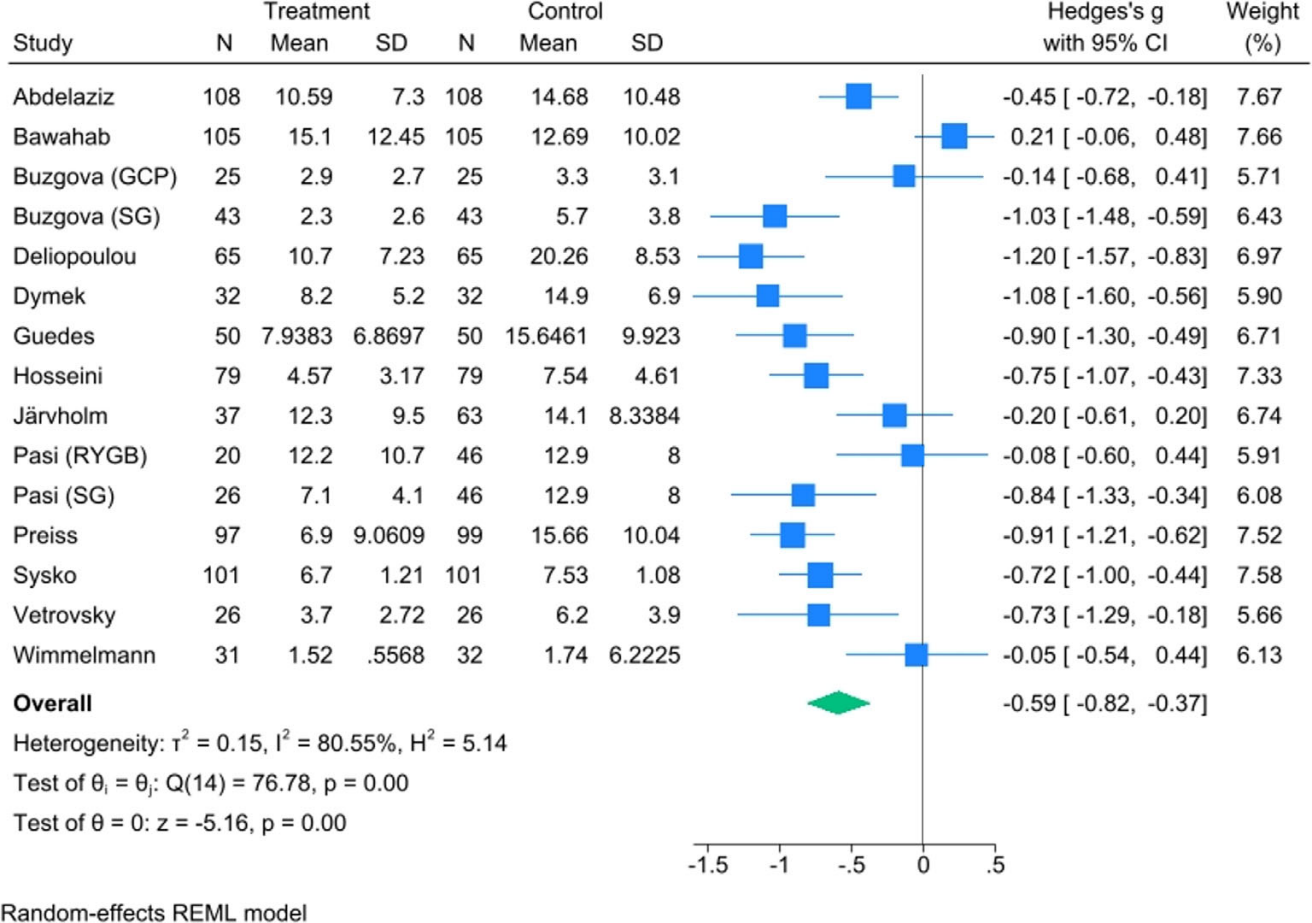
Forest plot of the effect of bariatric surgery on depression in the short‐term post‐surgery (0–4 months).

**FIGURE 3:**
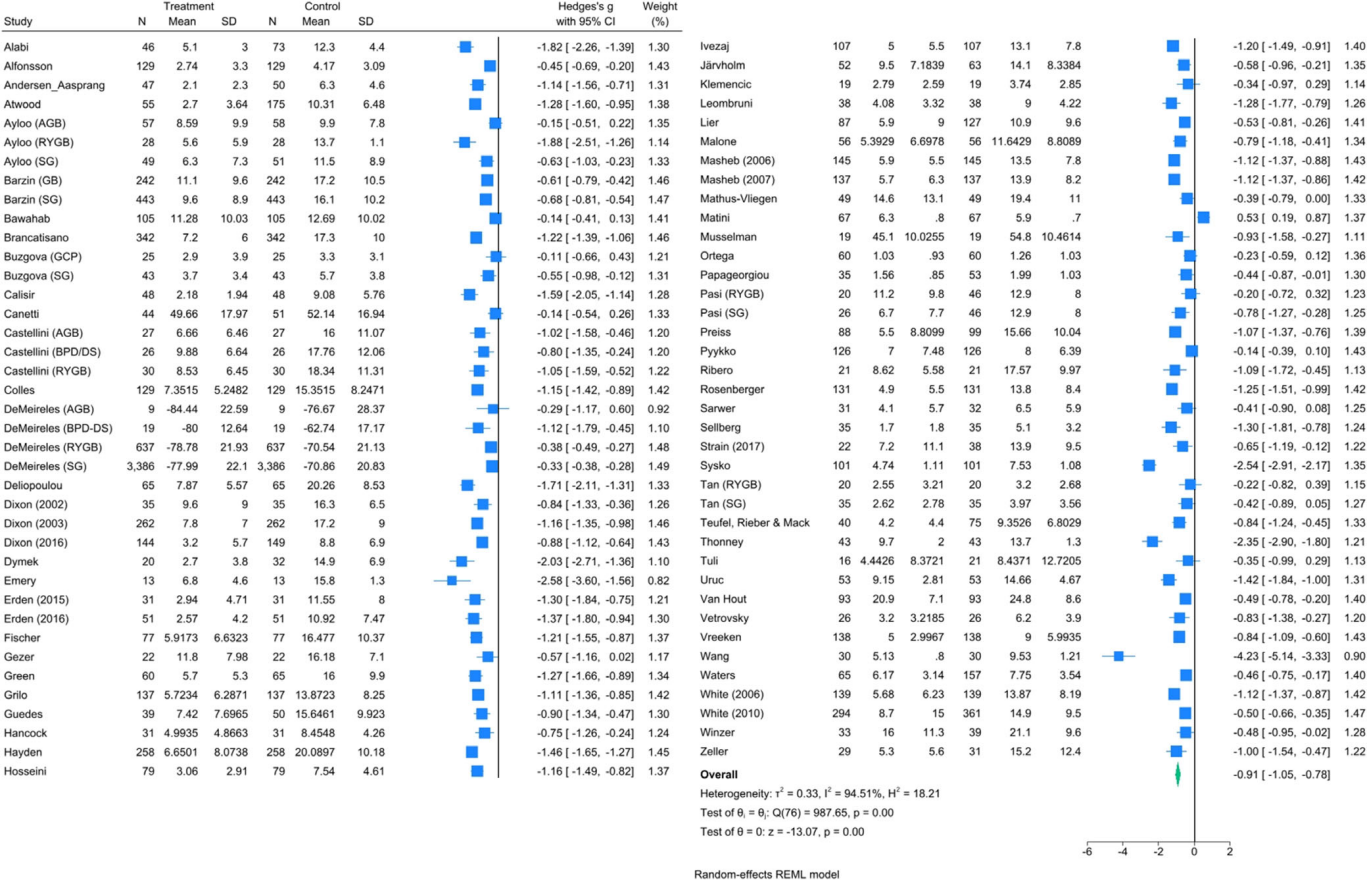
Forest plot of the effect of bariatric surgery on depression in the medium‐term post‐surgery (5–12 months).

**FIGURE 4:**
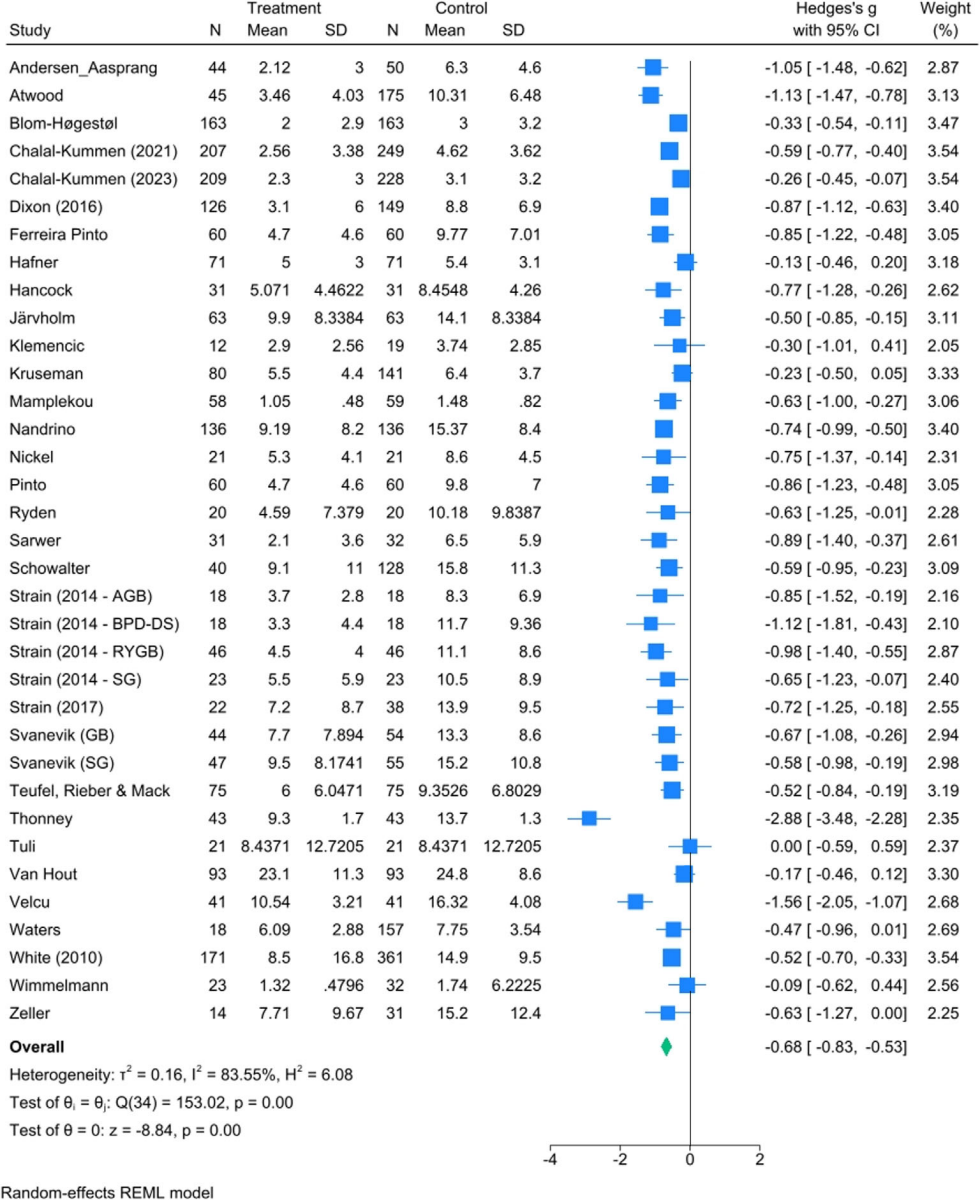
Forest plot of the effect of bariatric surgery on depression in the long‐term post‐surgery (> 12 months).

Meta‐analyses were conducted at each time point to assess changes in depressive symptoms over time. A meta‐analysis of 15 cohorts across 14 studies containing 928 patients with short‐term follow‐up indicated a reduction in depressive symptoms after MBS by an SMD of −0.6 (95% CI: −0.8, −0.4), indicating a moderate effect size, with high heterogeneity (*I* ^2^ = 80.6%) (Figure 2). At medium‐term follow‐up, a meta‐analysis of 77 cohorts across 72 studies containing 10,359 patients indicated a reduction in depressive symptoms after MBS by an SMD of −0.9 (95% CI: −1.1, −0.8), indicating a large effect size, with high heterogeneity (*I* ^2^ = 94.5%) (Figure 3). A meta‐analysis of 35 cohorts across 38 studies containing 2961 patients indicated a reduction in depressive symptoms at long‐term follow‐up by an SMD of −0.7 (95% CI: −0.9, −0.5), indicating a moderate effect size with high heterogeneity (*I* ^2^ = 83.6%) (Figure 4). These effects remained consistent in sensitivity analyses following the removal of studies consisting of adolescent or female‐only cohorts, non‐normally distributed data, and fair and/or poor‐quality studies (Tables S3–S5).

Meta‐regressions were conducted to assess differences in depression outcomes by MBS type, depression at baseline, and depression instrument used at short‐ (Table 2), medium‐ (Table 3), and long‐term (Table 4) follow‐up time points. Surgery Group explained none of the variance in depressive symptoms at short‐ (*p* = 0.633), medium‐ (*p* = 0.818), and long‐term (p = 0.509) follow‐up with high levels of heterogeneity (*I* ^2^ = 81.4%, 93.7% and 83.5%, respectively). Baseline depression accounted for 2.9%, 2.0%, and 1.1% of the variance in depressive symptoms at short‐ (*p* = 0. 221), medium‐ (*p* = 0. 147), and long‐term (*p* = 0. 168) follow‐up, with high levels of heterogeneity (*I* ^2^ = 80.1%, 93.1%, and 83.0%, respectively). Finally, Depression Instrument explained 7.1%, 6.1%, and 9.4% of the variance in depressive symptoms at short‐ (*p* = 0. 311), medium‐ (*p* = 0. 119), and long‐term (*p* = 0. 220) follow‐up with high levels of heterogeneity (*I* ^2^ = 79.5%, 94.1%, and 81.9%, respectively).

**TABLE 2:** Results of meta‐regressions with single covariates for depression from observational studies between baseline to short‐term post‐surgery (0–4 months).

	*k(n)* ^*a*^	SMD	(95% CI)	^β	(95% CI)^*b*^	*p*‐value	Adjusted *R* ^*2*^%	*I* ^*2*^% (Res)
Overall effect	15 (920)	−0.6	(−0.8, −0.4)	‐	‐	‐	‐	80.6%
*Surgery group*						0.6	0.0%	81.4%
Band (reference)	2 (200)	−0.8	(−1.0, −0.6)	‐	‐	‐		
Bypass	5 (199)	−0.4	(−0.8, −0.0)	0.4	(−0.3, 1.1)	0.3		
Sleeve	5 (381)	−0.5	(−1.0, −0.1)	0.3	(−0.4, 1.0)	0.5		
Malabsorptive		‐	‐	‐	‐	‐		
Other	3 (140)	−0.8	(−1.4, −0.2)	0.0	(−0.8, 0.8)	0.9		
Depression at baseline	15 (920)	‐	‐	0.0	(−0.1, 0.0)	0.2	2.9%	80.1%
*Depression instrument*						0.3	7.1%	79.5%
BDI (reference)	9 (610)	−0.7	(−1.0, −0.5)	‐	‐	‐		
DASS‐21	2 (184)	0.3	(1.2, 0.7)	0.2	(−0.1, 1.1)	0.2		
HADS‐D	3 (94)	−0.7	(1.2, −0.1)	0.8	(−0.5, 0.7)	0.8		
SCL‐90	1 (32)	0.0	(0.5, 0.4)	0.2	(−0.3, 1.6)	0.2		

*Note*: Adjusted *R* ^2^ is the percentage of between‐trial variance explained by the included covariates.

a K = number of comparisons; *n* = number of participants.

b Provides an estimate of the change in the SMD with each unit increase in the baseline standardized mean (calculated as the weighted average of baseline means divided by the pooled baseline standard deviation).

**TABLE 3:** Results of meta‐regressions with single covariates for depression from observation studies between baseline to medium‐term post‐surgery (5–12 months).

	*k(n)* ^*a*^	SMD	(95% CI)	^*β*	(95% CI)^*b*^	p‐value	Adjusted *R* ^*2*^%	*I* ^*2*^% (Res)
Overall effect	77 (10,385)	−0.9	(−1.1, −0.8)	‐	‐	‐	‐	94.5%
*Surgery group*						0.818	0.0%	93.7%
Band (reference)	13 (1549)	−1.0	(−1.3, −0.7)	‐	‐	‐		
Bypass	35 (3804)	−0.9	(−1.1, −0.7)	0.1	(−0.3, 0.5)	0.563		
Sleeve	17 (4505)	−1.0	(−1.4, −0.6)	0.1	(−0.4, 0.5)	0.807		
Malabsorptive	4 (133)	−0.9	(−1.2, −0.7)	0.1	(−0.6, 0.8)	0.800		
Other	8 (394)	−0.7	(−1.1, −0.3)	0.3	(−0.2, 0.9)	0.234		
Depression at baseline	77 (10,385)	‐	‐	0.0	(−0.0, 0.0)	0.147	2.0%	93.1%
*Depression instrument*						0.119	6.1%	93.0%
BDI (reference)	45 (4748)	−1.1	(−1.2, −0.9)	‐	‐	‐		
BODY‐Q	4 (4051)	−0.3	(−0.4, −0.3)	0.6	(−0.0, 1.2)	0.557		
CED‐D	2 (175)	−0.2	(−0.4, 0.0)	0.8	(−0.0, 1.6)	0.805		
DASS‐21	2 (184)	−0.6	(−1.6, 0.4)	0.4	(−0.4, 1.2)	0.428		
HADS‐D	9 (394)	−0.6	(−0.9, −0.4)	0.4	(−0.0, 0.9)	0.428		
HAM‐D	3 (145)	−1.7	(−4.4, 0.9)	−0.4	(−1.1, 0.4)	0.327		
HIS‐GWB	1 (157)	−0.5	(−0.8, −0.2)	0.6	(−0.5, 1.7)	0.609		
MHI	1 (51)	−0.1	(−0.5, 0.3)	0.9	(−0.2, 2.1)	0.928		
PHQ‐9	2 (215)	−1.1	(−1.5, −0.6)	0.0	(−0.8, 0.8)	0.004		
SCL‐90	4 (227)	−0.5	(−0.7, −0.2)	0.5	(−0.1, 1.1)	0.528		
YSR/ASR	1 (19)	−0.3	(−1.0, 0.3)	0.7	(−0.5, 2.0)	0.727		
ZDRS	1 (19)	−0.9	(−1.6, −0.3)	0.1	(−1.1, 1.4)	0.142		

*Note*: Adjusted *R* ^2^ is the percentage of between‐trial variance explained by the included covariates.

a K = number of comparisons; *n* = number of participants.

b Provides an estimate of the change in the SMD with each unit increase in the baseline standardized mean (calculated as the weighted average of baseline means divided by the pooled baseline standard deviation).

**TABLE 4:** Results of meta‐regressions with single covariates for depression from observation studies between baseline to long‐term post‐surgery (> 12 months).

	*k(n)* ^*a*^	SMD	(95% CI)	^*β*	(95% CI)^*b*^	*p*‐value	Adjusted *R* ^*2*^%	*I* ^*2*^% (Res)
Overall effect	35 (2961)	−0.7	(−0.8, −0.5)	‐	‐	‐	‐	83.6%
*Surgery group*						0.509	0.0%	83.5%
Band (reference)	6 (483)	−0.8	(−0.9, −0.7)	‐	‐	‐		
Bypass	17 (1758)	−0.7	(−1.0, −0.4)	0.0	(−0.4, 0.5)	0.860		
Sleeve	5 (423)	−0.5	(−0.7, −0.4)	0.3	(−0.3, 0.8)	0.325		
Malabsorptive	3 (106)	−1.0	(−1.3, −0.7)	−0.2	(−0.9, 0.5)	0.563		
Other	4 (191)	−0.4	(−0.7, −0.1)	0.3	(−0.3, 0.9)	0.274		
Depression at baseline	35 (2961)	‐	‐	0.0	(−0.1, 0.0)	0.168	1.1%	83.0%
*Depression instrument*						0.220	9.4%	81.9%
BDI (reference)	20 (1397)	−0.8	(−1.1, −0.6)	‐	‐	‐		
CCEI	1 (71)	−0.1	(−0.5, 0.2)	0.7	(−0.1, 1.5)	0.098		
HADS‐D	7 (883)	−0.5	(−0.7, −0.3)	0.3	(−0.1, 0.7)	−0.067		
HIS‐GWB	1 (157)	−0.5	(−1.0, 0.0)	0.4	(−0.5, 1.3)	−0.550		
PHQ‐9	2 (250)	−0.8	(−1.4, −0.2)	0.0	(−0.6, 0.6)	−0.592		
SCL‐90	3 (184)	−0.3	(−0.6, 0.0)	0.5	(0.0, 1.1)	0.006		
YSR/ASR	1 (19)	−0.3	(−1.0, 0.4)	0.5	(−0.5, 1.6)	−0.507		

*Note*: Adjusted *R* ^2^ is the percentage of between‐trial variance explained by the included covariates.

a K = number of comparisons; *n* = number of participants.

b Provides an estimate of the change in the SMD with each unit increase in the baseline standardized mean (calculated as the weighted average of baseline means divided by the pooled baseline standard deviation).

## 4 DISCUSSION

The primary finding of this meta‐analysis of 82 studies was that overall self‐reported depressive symptoms improved following MBS with a moderate to high effect size at all post‐operative phases, peaking in the medium‐term. Qualitative analysis indicated an overall reduction in depressive symptoms following all types of bariatric procedures, at each time point. However, it was also identified that many post‐surgical patients demonstrate higher than normal levels of depression as compared to the general population and that a subset of patients experienced a worsening or new indication of depression and associated mental health concerns following bariatric procedures. A variety of factors were associated with changes in depressive symptoms including weight loss, eating‐related behaviors, and existing mental health concerns, as well as multiple demographic factors and concurrent medical conditions.

In attempting to identify sources of variance in this patient population, we investigated procedure type, baseline depression scores, and the instrument used. Despite qualitative evidence in the literature, our meta‐analysis found no evidence that surgery type, baseline depression, or depression instrument explained the variance in depressive symptoms at any time point post‐surgery. The broad inclusion criteria used in this review have facilitated a comprehensive qualitative and quantitative analysis of depression outcomes over time. The inclusion of data that distinguishes between surgery types and across time points has allowed us to conduct subgroup analyses at multiple follow‐up periods, avoiding collapsing data across surgery groups or time points. This has allowed for specific analysis of differences in depressive symptoms by surgery type at each time point. In line with our review, previous reviews have repeatedly found an overall reduction in depression and evidence that initial improvements deteriorate over time. Previous reviews have also been unable to identify risk factors or mediators of depression outcomes in this patient cohort and have identified high levels of heterogeneity.^11^, ^170^, ^171^, ^172^, ^173^, ^174^, ^175^ These cumulative results highlight the diversity of patient experience post‐surgery and suggest that any trends in depression or other patient outcomes present in patient subgroups may be lost in meta‐analyses as results tend to regress towards the mean. As such, the results of this review were unable to identify any sources of heterogeneity for the bariatric population or to identify any potential risk factor for adverse mental health effects experienced by some patients. The heterogeneity of patient outcomes is of particular interest within the field as understanding the mechanisms by which bariatric procedures work, not only to generate weight loss but also in their impacts on quality of life and psychosocial well‐being. This may facilitate improved and personalized patient care by ensuring patients are appropriately informed of the potential psychiatric outcomes of surgery, providing appropriate care to those patients experiencing poor outcomes, and preventing any additional adverse outcomes related to mental health including disordered eating behaviors and reduced weight loss.

Overall, this review is limited by a high number of poor‐ to fair‐quality studies, small sample sizes, short‐ follow‐up, and high attrition rates. In addition, many studies did not account for missing data in their analyses which may bias results, particularly given the observation that patients who do more poorly are less inclined to attend follow‐up.^97^ Despite the comprehensive search strategy, this review may be subject to selective reporting bias. Depression is often a secondary outcome in bariatric studies and may not have been mentioned in the title and/or abstract. The exclusion of non‐English‐language studies and the study population consisting of primarily middle‐aged Caucasian women, from high socioeconomic countries, precludes the results from being generalized to other settings or populations. Although baseline depression and depression instrument used may explain some of the variance, there remains a significant amount of heterogeneity at all time points. Studies that included a control group to account for this were few but generally suggested bariatric procedures have a significant impact on depressive symptoms within a population when compared to both healthy controls and individuals living with obesity.^80^, ^92^, ^116^, ^132^, ^133^, ^140^, ^144^, ^150^, ^155^ Despite this, the combined results of this review are hampered by the inability to identify risk factors in the groups of patients who improve compared to those who experience worsening depressive symptoms post‐surgery, resulting in high levels of heterogeneity and the regression of depressive symptoms towards the mean. Prospective studies that define patient trajectories by their depression outcomes, as opposed to their weight‐related outcomes, may prove useful in identifying risk factors for worsening depressive symptoms post‐surgery so that those patients experiencing adverse mental health may be better identified.^155^, ^176^, ^177^

## 5 CONCLUSION

Patient‐reported depressive symptoms generally improve following all types of MBS at short‐, medium‐, and long‐term follow‐up. Improvements are observed in the short‐term post‐operative phase, peaking in the medium‐term with depressive symptoms returning towards pre‐surgical levels at long‐term follow‐up. There remains a subset of patients experiencing a decline in mental health post‐surgery; however, these subsets cannot be captured in meta‐analyses and contribute to high levels of heterogeneity in the sample. The significant variance within the results cannot be explained by surgery type, baseline depression, or depression instrument used across studies. As such, although there is a trend towards improved depression post‐operatively which is lost over time, no conclusions can be made as to the positive or negative influence of bariatric procedures on mental health outcomes. Prospective studies assessing these outcomes with consistent, validated measures incorporating non‐surgical control groups are necessary to further elucidate the relationship among obesity, MBS, and depression so that researchers and clinicians can better identify and care for at‐risk patients.

## CONFLICT OF INTEREST STATEMENT

W.A. Brown reports grants from Novo Nordisk and Myerton Australia, and personal fees from GORE, Novo Nordisk, Pfizer, and Merck Sharp & Dohme for lectures and advisory boards. I.D. Caterson reports grants from Eli Lilly and Boehringer Ingelheim for clinical trials. He is a past‐president of World Obesity and on the board of Obesity Australia. P. Sumithran reports co‐authorship of manuscripts with medical writing assistance from Novo Nordisk and Eli Lilly and receiving payment to her academic institution from Eli Lilly for participation in advisory meetings.

## Supporting information


**Table S1** Example Search Strategy.
**Table S2** Quality assessment of included articles according to the National Heart, Long, and Blood Institute quality assessment tool for before‐after (pre‐post) studies with no control group.
**Table S3** Effect of sensitivity analysis on meta‐analysis between baseline to short‐term post‐surgery (0–4 months).
**Table S4** Effect of sensitivity analysis on meta‐analysis between baseline to Medium‐Term post‐surgery (5–12 months).
**Table S5** Effect of sensitivity analysis on meta‐analysis between baseline to long‐term post‐surgery (> 12 months).
