# Supplementary material for: Depressive symptoms at short‐, medium‐, and long‐term follow‐up after bariatric surgical procedures: A systematic review and meta‐analysis
Source: Obes Rev. 2025 Apr 13;26(8):e13927. doi: 10.1111/obr.13927 (PMC12246895; doi:10.1111/obr.13927)
Supplement: Supplementary file 1 — Table S1 Example Search Strategy. Table S2 Quality assessment of included articles according to the National Heart, Long, and Blood Institute quality assessment tool for before‐after (pre‐post) studies with no control group. Table S3 Effect of sensitivity analysis on meta‐analysis between baseline to short‐term post‐surgery (0–4 months). Table S4 Effect of sensitivity analysis on meta‐analysis between baseline to Medium‐Term post‐surgery (5–12 months). Table S5 Effect of sensitivity analysis on meta‐analysis between baseline to long‐term post‐surgery (> 12 months). [file OBR-26-e13927-s001.pdf]

Depressive Symptoms at Short, Medium and Long-Term Follow-Up After Bariatric Surgical Procedures: A Systematic Review & Meta-Analysis

Supplementary Material

Dr Alyssa J. Budin[1], Professor Wendy A. Brown[1,2], Associate Professor Andrew D. MacCormick[3,4], Professor Ian Caterson[5,6], & Associate Professor Priya Sumithran*[1,7].

[1] Department of Surgery, School of Translational Medicine, Monash University, The Alfred Centre, Melbourne, VIC, Australia
[2] Alfred Health, The Alfred Centre, Melbourne, VIC, Australia
[3] Department of Surgery, The University of Auckland, Auckland, New Zealand
[4] Te Whatu Ora Counties Manukau Otahuhu, Auckland, New Zealand
[5] The Boden Initiative, Charles Perkins Centre, The University of Sydney, Camperdown, NSW, Australia
[6] Department of Endocrinology, Royal Prince Alfred Hospital, Camperdown, NSW, Australia
[7] Department of Endocrinology and Diabetes, Alfred Health Melbourne, VIC, Australia

***Correspondence to:**

Priya Sumithran
priya.sumithran@monash.edu
(03) 9903 8939
Monash University
Level 6, The Alfred Centre
99 Commercial Rd, Melbourne, VIC, 3004
Australia

Keywords: Bariatric Surgery, Depression, Patient-Reported Measures, Psychosocial Health

Supplementary Table S1 Example Search Strategy

Ovid MEDLINE(R) and Epub Ahead of Print, In-Process, In-Data-Review & Other Non-Indexed Citations, Daily and Versions 1946 to January 17, 2024

#	Searches	Results
1	bariatrics/ or bariatric surgery/ or anastomosis, roux-en-y/ or biliopancreatic diversion/ or gastrectomy/ or gastric bypass/ or gastroplasty/ or jejunoileal bypass/ or gastric balloon/	70,892
2	(Bariatric* or sleeve gastrectomy or duodenal switch or biliopancreatic diversion or lap-band or laparoscopic band* or Roux-en-y or aspire assist or intragastric balloon).ti,ab.	39,227
3	(bypass adj2 (ileal or jejuno-ileal or duodenal-ileal or duodeno-jejunal or gastroileal or jejun* or duod*)).ti,ab.	2,196
4	(gastric adj2 (band* or balloon* or bypass or sleeve)).ti,ab.	17,785
5	(gastroplasty adj3 (vertical or sleeve)).ti,ab.	1,313
6	(surg* adj2 (weight or obesity or metabolic)).ti,ab.	8,391
7	1 or 2 or 3 or 4 or 5 or 6	86,485
8	depression/ or mood disorders/ or depressive disorder/ or depressive disorder, major/ or depressive disorder, treatment-resistant/ or mental health/	323,749
9	(depress* or antidepress* or mental health).ti,ab.	773,484
10	(psycho* or psychi*).ti.	383,092
11	(disorder* adj2 (mood or psych*)).ti,ab.	98,886
12	8 or 9 or 10 or 11	1,155,211
13	obesity/ or obesity, abdominal/ or obesity, morbid/ or overweight/ or weight loss/ or body mass index/ or body weight/	534,287
14	(body adj2 (weight or mass)).ti,ab.	507,073
15	(obes* or BMI or overweight or weight loss or weight reduc*).ti,ab.	592,448
16	13 or 14 or 15	1,073,983
17	7 and 12 and 16	1,819
18	exp animals/ not humans.sh.	5,187,621
19	17 not 18	1,800
20	limit 19 to english language	1,723
21	(case reports or clinical conference or comment or editorial or guideline or letter or newspaper article or published erratum).pt.	4,551,512
22	20 not 21	1,630

Supplementary Table S2 Quality assessment of included articles according to the National Heart, Long, and Blood Institute quality assessment tool for before-after (pre-post) studies with no control group

Ref	Study	Reference	1	2	3	4	5	6	7	8	9	10	11	Overall Rating (of 11)
26	4	Aasprang (2013)	Y	Y	Y	Y	Y	Y	Y	N/A	Y	Y	Y	Good (10)
27	1	Abdelaziz (2023)	Y	Y	Y	Y	N	Y	Y	N/A	Y	Y	Y	Good (9)
28	2	Alabi (2018)	Y	Y	Y	Y	N	N	Y	N/A	N	Y	Y	Fair (7)
29	3	Alfonsson (2014)	Y	N	Y	N	Y	N	Y	N/A	Y	Y	Y	Fair (7)
30	4	Aasprang (2013)	Y	Y	Y	Y	Y	Y	Y	N/A	Y	Y	Y	Good (10)
31	5	Atwood (2021)	Y	N	Y	Y	N	Y	Y	N/A	N	N	Y	Fair (6)
32	6	Ayloo (2015)	Y	Y	N	N	N	Y	Y	?	N	N	Y	Poor (5)
33	7	Barzin (2020)	Y	Y	Y	Y	Y	Y	Y	?	Y	Y	Y	Good (10)
34	8	Bawahab (2022)	Y	Y	Y	Y	N	Y	Y	N/A	Y	Y	Y	Good (9)
35	9	Blom-Høgestøl (2023)	Y	N	?	Y	N	Y	Y	N/A	Y	Y	Y	Fair (7)
36	10	Brancatisano (2008)	Y	Y	Y	Y	Y	Y	N	N/A	N	Y	N	Fair (7)
37	11	Buzgova (2016)	Y	Y	Y	?	N	Y	Y	?	Y	Y	Y	Good (8)
38	12	Calisir (2020)	Y	Y	Y	N	N	N	Y	N/A	Y	Y	Y	Fair (7)
39	13	Canetti (2016)	Y	N	Y	Y	Y	Y	Y	N/A	Y	N	Y	Fair (8)
40	14	Castellini (2014)	Y	Y	Y	Y	N	Y	Y	Y	Y	Y	Y	Good (10)
41	15	Chahal-Kummen (2021)	Y	Y	?	?	N	Y	Y	N/A	Y	Y	Y	Good (7)
42	15	Chahal-Kummen (2023)	Y	Y	?	Y	N	Y	Y	N	Y	Y	Y	Good (8)
43	16	Colles (2008)	Y	Y	Y	Y	N	Y	Y	N/A	N	Y	Y	Fair (8)
44	17	Deliopoulou (2013)	Y	Y	Y	Y	N	Y	N	N/A	Y	N	N	Poor (6)
45	18	deMeireles (2020)	Y	Y	Y	Y	Y	N	Y	?	N	Y	Y	Good (8)
46	19	Dixon (2002)	Y	Y	N	Y	N	Y	Y	N/A	N	N	N	Poor (5)
47	20	Dixon (2003)	Y	Y	Y	Y	N	Y	Y	N/A	Y	Y	Y	Fair (9)
48	21	Dixon (2016)	Y	Y	Y	Y	Y	Y	Y	N/A	N	Y	Y	Good (9)
49	22	Dymek (2001)	Y	Y	Y	Y	N	Y	Y	N/A	N	Y	Y	Fair (8)
50	23	Emery (2007)	Y	N	N	?	N	Y	Y	N/A	Y	Y	Y	Poor (6)
51	24	Erden (2015)	Y	Y	Y	N	N	Y	Y	N/A	N	Y	Y	Fair (7)
52	25	Erden (2016)	Y	Y	Y	Y	N	Y	Y	Y	Y	Y	Y	Good (10)
53	26	Ferreira (2017)	Y	Y	Y	Y	N	Y	N	N/A	Y	Y	Y	Fair (8)
54	27	Fischer (2007)	Y	Y	N	Y	N	Y	N	N/A	Y	N	N	Poor (5)
55	28	Gezer (2023)	Y	Y	Y	Y	N	Y	Y	N/A	N	Y	Y	Fair (8)
56	29	Green (2004)	Y	Y	Y	?	N	Y	Y	N/A	Y	Y	Y	Fair (8)
57	30	Grilo (2006)	Y	N	?	?	N	N	Y	N/A	Y	N	Y	Poor (4)
58	31	Guedes (2016)	Y	Y	N	Y	N	Y	Y	N/A	N	Y	Y	Fair (7)
59	32	Hafner (1990)	Y	N	Y	?	N	N	Y	N	Y	Y	Y	Fair (6)
60	33	Hancock (2018)	Y	Y	N	Y	N	?	Y	N/A	Y	Y	Y	Fair (7)
61	34	Hayden (2011)	Y	Y	Y	Y	N	?	Y	N/A	Y	N	Y	Fair (7)
62	35	Hosseini (2023)	Y	Y	Y	N	N	Y	Y	N/A	Y	Y	Y	Fair (8)
63	36	Ivezaj (2015)	Y	N	?	?	N	?	Y	N/A	Y	N	Y	Fair (4)
64	37	Jarvholm (2011)	Y	Y	Y	N	N	Y	Y	N/A	Y	Y	Y	Good (8)
65	37	Jarvholm (2015)	Y	Y	Y	Y	N	Y	Y	N/A	N	Y	Y	Good (8)
66	38	Klemencic (2021)	Y	Y	Y	?	N	Y	Y	N/A	N	Y	Y	Fair (7)
67	39	Kruseman (2010)	Y	Y	Y	N	N	Y	N	Y	Y	Y	Y	Good (8)
68	40	Leombruni (2007)	Y	Y	Y	N	N	Y	Y	N/A	Y	Y	Y	Good (8)
69	41	Lier (2013)	Y	N	Y	Y	N	Y	Y	N/A	N	Y	Y	Good (7)
70	68	Mack (2016)	Y	Y	N	N	N	?	Y	N/A	N	Y	Y	Poor (5)
71	42	Malone (2004)	Y	Y	Y	?	N	?	Y	N/A	N	Y	Y	Fair (6)
72	43	Mamplekou (2005)	Y	Y	Y	?	N	Y	Y	N/A	Y	Y	Y	Fair (8)
73	44	Masheb (2006)	Y	N	?	?	N	?	Y	N/A	Y	Y	Y	Fair (5)
74	45	Masheb (2007)	Y	Y	Y	?	N	?	Y	N/A	Y	Y	Y	Good (7)
75	46	Mathus-Vliegen (2004)	Y	Y	Y	Y	N	Y	Y	?	Y	Y	Y	Good (9)
76	47	Matini (2014)	Y	Y	Y	N	N	?	Y	?	Y	Y	Y	Fair (7)
77	48	Musselman (2019)	Y	Y	N	Y	N	Y	Y	N/A	Y	Y	Y	Fair (8)
78	49	Nandrino (2020)	Y	Y	Y	Y	N	Y	Y	N/A	Y	Y	Y	Fair (9)
79	50	Nickel (2005)	Y	Y	N	Y	N	Y	Y	?	Y	Y	Y	Poor (8)
80	50	Nickel (2007)	Y	?	N	?	N	Y	Y	?	?	N	Y	Poor (4)
81	51	Ortega (2012)	Y	N	Y	?	N	Y	Y	N/A	Y	Y	Y	Fair (7)

82	52	Papageorgiou (2002)	Y	N	?	?	N	Y	Y	N/A	N	Y	Y	Fair (5)
83	53	Pasi (2023)	Y	Y	Y	N	Y	?	Y	?	Y	Y	Y	Good (8)
84	54	Pinto (2017)	Y	Y	Y	Y	N	Y	Y	N/A	Y	Y	Y	Good (9)
85	55	Preiss (2018)	Y	Y	Y	Y	N	Y	Y	N/A	Y	Y	Y	Fair (9)
86	56	Pyykko (2021)	Y	Y	Y	Y	N	N	Y	?	Y	Y	Y	Fair (8)
87	57	Ribeiro (2022)	Y	Y	N	N	N	Y	Y	N/A	N	Y	Y	Poor (6)
88	68	Rieber (2013)	Y	N	?	?	N	Y	Y	?	N	Y	Y	Fair (5)
89	58	Rosenberger (2011)	Y	N	Y	N	N	Y	Y	N/A	N	Y	Y	Fair (6)
90	59	Ryden (1996)	Y	N	Y	Y	N	Y	Y	N/A	Y	Y	Y	Fair (8)
91	60	Sarwer (2015)	Y	Y	N	Y	N	Y	Y	N/A	Y	Y	Y	Fair (8)
92	61	Schowalter (2008)	Y	Y	Y	Y	N	Y	Y	N/A	N	Y	Y	Fair (8)
93	62	Sellberg (2018)	Y	Y	N	?	N	?	Y	N/A	N	Y	Y	Fair (5)
94	63	Strain (2014)	Y	N	?	?	N	?	Y	?	Y	Y	Y	Poor (5)
95	64	Strain (2017)	Y	Y	Y	Y	N	Y	Y	N/A	N	N	Y	Poor (7)
96	65	Svanevik (2023)	Y	Y	Y	N	N	Y	Y	Y	Y	N	Y	Fair (8)
97	66	Sysko (2012)	Y	Y	Y	Y	N	Y	Y	N/A	Y	N	Y	Fair (8)
98	67	Tan (2021)	Y	Y	Y	Y	N	Y	Y	N	N	N	Y	Fair (7)
99	68	Teufel (2012)	Y	Y	Y	Y	N	Y	Y	?	Y	Y	Y	Fair (9)
100	69	Thonney (2010)	Y	N	N	?	N	?	Y	N/A	Y	Y	Y	Poor (5)
101	70	Tuli (2024)	Y	Y	Y	?	N	Y	Y	?	Y	N	Y	Fair (7)
102	71	Uruc (2016)	Y	Y	N	?	N	Y	Y	N/A	Y	Y	Y	Fair (7)
103	72	VanHout (2008)	Y	Y	Y	Y	N	Y	Y	N/A	N	Y	Y	Fair (8)
104	73	Velcu (2005)	Y	Y	Y	Y	N	Y	Y	N	N	Y	Y	Fair (8)
105	74	Vetrovsky (2021)	Y	Y	Y	Y	N	Y	Y	N	Y	N	Y	Fair (8)
106	75	Vreeken (2023)	Y	Y	Y	Y	N	Y	Y	N/A	Y	Y	Y	Good (9)
107	76	Wang (2022)	Y	Y	Y	Y	N	Y	Y	N/A	Y	Y	Y	Good (9)
108	77	Waters (1991)	Y	Y	Y	N	N	Y	Y	N/A	N	?	Y	Fair (6)
109	78	White (2006)	Y	N	?	?	N	Y	Y	N/A	Y	N	Y	Poor (5)
110	79	White (2010)	Y	N	?	?	N	Y	Y	N/A	N	N	Y	Poor (4)
111	80	Wimmelmann (2016)	Y	Y	Y	Y	N	Y	Y	N/A	N	Y	Y	Fair (8)
112	81	Winzer (2020)	Y	Y	Y	N	N	Y	Y	?	Y	Y	Y	Good (8)
113	82	Zeller (2009)	Y	Y	Y	Y	N	Y	Y	N/A	Y	N	Y	Good (8)
114	82	Zeller (2011)	Y	Y	Y	Y	N	Y	Y	N/A	Y	N	Y	Good (8)
115	82	Zeller (2017)	Y	Y	Y	Y	N	Y	Y	N/A	Y	N	Y	Good (8)

1. Was the study question or objective clearly stated?
2. Were eligibility/selection criteria for the study population pre-specified and described clearly?
3. Were the participants in the study representative of those who would be eligible for the test/service/intervention in the general or clinical population of interest?
4. Were all eligible participants that met the pre-specified entry criteria enrolled?
5. Was the sample size sufficiently large to provide confidence in the findings?
6. Was the test/service/intervention clearly described and delivered consistently across the study population?
7. Were the outcome measures pre-specified, clearly defined, valid, reliable, and assessed consistently across all study participants?
8. Were the people assessing the outcomes blinded to the participants' exposures/interventions?
9. Was the loss to follow-up after baseline 20 per cent or less? Were those lost to follow-up accounted for in the analysis?
10. Did the statistical methods examine changes in outcome measures from before to after the intervention? Were statistical tests done that provided P-values for the pre-to-post changes?
11. Were outcome measures of interest taken multiple times before the intervention and multiple times after the intervention (did they use an interrupted time-series design)?

Y, yes; N, no; ?, not reported or cannot be determined; N/A, not applicable

Supplementary Table S3 Effect of sensitivity analysis on meta-analysis between baseline to short-term post-surgery (0 – 4 months)

Outcome of Interest	No. of studies	Total no. of patients		SMD	(95% CI)[b]	I^2%
		Pre-Surgery	*Post-Surgery*			
**Age**						
Removal of studies with only age ≤ 18 years	13	756	707	-0.611	(-0.866, -0.357)	81.6%
**Sex**						
Removal of female-only studies	14	855	780	-0.546	(-0.769, -0.323)	78.5%
**Quality Assessment**						
Good quality studies only	7	436	364	-0.350	(-0.682, -0.018)	79.8%
Good & fair quality studies only	14	855	780	-0.546	(-0.769, -0.323)	78.5%
**Questionnaire Used**						
BDI	9	610	536	-0.713	(-0.951, -0.474)	72.5%
DASS-21	2	184	184	-0.263	(-1.204, 0.677)	95.0%
HADS-D	3	94	94	-0.651	(-1.173, -0.128)	67.8%
SCL-90	1	32	31	-0.049	(-0.537, 0.439)	-
**One-sample removed analysis**						
Abdelaziz *et al.*	14	812	737	-0.604	(-0.846, -0.361)	80.9%
Bawahab *et al.*	14	815	740	-0.663	(-0.858, -0.467)	70.3%
Buzgova *et al.* - GCP	14	877	802	-0.620	(-0.852, -0.388)	81.2%
Buzgova *et al.* - SG	14	895	820	-0.562	(-0.794, -0.329)	80.8%
Deliopoulou *et al.*	14	855	780	-0.546	(-0.769, -0.323)	78.5%
Dymek *et al.*	14	888	813	-0.561	(-0.792, -0.33)	80.9%
Guedes *et al.*	14	870	795	-0.57	(-0.807, -0.333)	81.4%
Hosseini *et al.*	14	841	766	-0.58	(-0.821, -0.338)	81.4%
Jarvholm *et al.*	14	857	808	-0.62	(-0.855, -0.386)	80.9%
Pasi *et al.* - RYGB	14	874	825	-0.624	(-0.854, -0.394)	80.8%
Pasi *et al.* - SG	14	874	819	-0.576	(-0.814, -0.339)	81.9%
Preiss *et al.*	14	821	748	-0.566	(-0.803, -0.329)	80.4%
Sysko *et al.*	14	819	744	-0.582	(-0.824, -0.339)	81.1%
Vetrovsky *et al.*	14	894	819	-0.584	(-0.822, -0.345)	82.2%
Wimmelmann *et al.*	14	888	814	-0.627	(-0.856, -0.399)	80.4%
**Non-normally distributed studies**						
Removal of all studies where mean(3 SD) included negative values	1	101	101	-0.721	(-1.005, -0.437)	-

Supplementary Table S4 Effect of sensitivity analysis on meta-analysis between baseline to Medium-Term post-surgery (5 - 12 months)

Outcome of Interest	No. of studies	Total no. of patients *Pre-Surgery*	Total no. of patients *Post-Surgery*	SMD	(95% CI)[b]	I^2%
**Age**						
Removal of studies with only age ≤ 18 years	73	10,343	9,757	-0.899	(-1.034, -0.765)	94.2%
**Sex**						
Removal of female-only studies	69	10,277	9,678	-0.847	(-0.985, -0.709)	94.4%
**Quality Assessment**						
Good quality studies only	26	5,994	5,881	-0.797	(-1.029, -0.565)	95.8%
Good & fair quality studies only	64	9,491	8,978	-0.865	(-1.012, -0.719)	94.5%
**Questionnaire Used**						
BDI	47	4,920	4,561	-1.065	(-1.21, -0.919)	89.9%
BODY-Q	4	4,051	4,051	-0.343	(-0.387, -0.299)	0.0%
CED-D	2	175	175	-0.218	(-0.443, 0.006)	9.4%
DASS-21	2	184	184	-0.643	(-1.639, 0.353)	95.3%
HADS-D	9	394	391	-0.642	(-0.892, -0.392)	63.2%
HAM-D	3	145	145	-1.739	(-4.426, 0.947)	98.8%
HIS-GWB	1	157	65	-0.459	(-0.751, -0.168)	-
MHI	1	51	44	-0.141	(-0.542, 0.259)	-
PHQ-9	2	215	95	-1.076	(-1.503, -0.648)	64.3%
SCL-90	4	227	209	-0.475	(-0.706, -0.244)	26.9%
YSR/ASR	1	19	19	-0.342	(-0.969, 0.286)	-
ZDRS	1	19	19	-0.927	(-1.583, -0.27)	-
**One-sample removed analysis**						
Abdelaziz *et al.*	14	812	737	-0.604	(-0.846, -0.361)	80.9%
Alabi *et al.*	76	10,484	9,912	-0.902	(-1.038, -0.765)	94.4%
Alfonsson *et al.*	76	10,428	9,829	-0.921	(-1.059, -0.782)	94.5%
Andersen *et al.* & Aasprang *et al.*	76	10,507	9,911	-0.911	(-1.05, -0.772)	94.6%
Atwood *et al.*	76	10,382	9,903	-0.909	(-1.048, -0.77)	94.6%
Ayloo *et al.* - AGB	76	10,499	9,901	-0.924	(-1.062, -0.787)	94.5%
Ayloo *et al.* - RYGB	76	10,529	9,930	-0.902	(-1.039, -0.766)	94.5%
Ayloo *et al.* - SG	76	10,506	9,909	-0.918	(-1.057, -0.779)	94.6%
Barzin *et al.* - GB	76	10,315	9,716	-0.919	(-1.058, -0.78)	94.5%
Barzin *et al.* - SG	76	10,114	9,515	-0.918	(-1.057, -0.779)	94.4%
Bawahab *et al.*	76	10,452	9,853	-0.925	(-1.062, -0.788)	94.4%
Brancatisano *et al.*	76	10,215	9,616	-0.910	(-1.048, -0.771)	94.4%
Buzgova *et al.* - GCP	76	10,532	9,933	-0.924	(-1.061, -0.786)	94.5%
Buzgova *et al.* - SG	76	10,514	9,915	-0.919	(-1.058, -0.78)	94.6%
Calisir *et al.*	76	10,509	9,910	-0.905	(-1.043, -0.767)	94.5%
Canetti *et al.*	76	10,506	9,914	-0.924	(-1.061, -0.787)	94.5%
Castellini *et al.* - AGB	76	10,530	9,931	-0.913	(-1.052, -0.774)	94.6%
Castellini *et al.* - BPD-DS	76	10,531	9,932	-0.916	(-1.054, -0.777)	94.6%
Castellini *et al.* - RYGB	76	10,527	9,928	-0.912	(-1.051, -0.774)	94.6%
Colles *et al.*	76	10,428	9,829	-0.911	(-1.05, -0.772)	94.6%
Deliopoulou *et al.*	76	10,492	9,893	-0.920	(-1.058, -0.782)	94.5%

deMeireles *et al.* - AGB	76	10,548	9,949	-0.912	(-1.05, -0.773)	94.6%
deMeireles *et al.* - BPD-DS	76	10,538	9,939	-0.922	(-1.06, -0.784)	94.6%
deMeireles *et al.* - RYGB	76	9,920	9,321	-0.923	(-1.061, -0.785)	94.1%
deMeireles *et al.* - SG	76	7,171	6,572	-0.903	(-1.04, -0.766)	92.7%
Dixon *et al.* (2002)	76	10,522	9,923	-0.915	(-1.054, -0.776)	94.6%
Dixon *et al.* (2003)	76	10,295	9,696	-0.910	(-1.049, -0.772)	94.5%
Dixon *et al.* (2016)	76	10,408	9,814	-0.915	(-1.054, -0.776)	94.6%
Dymek *et al.*	76	10,525	9,938	-0.901	(-1.037, -0.765)	94.4%
Emery *et al.*	76	10,544	9,945	-0.900	(-1.035, -0.765)	94.4%
Erden *et al.* (2015)	76	10,526	9,927	-0.909	(-1.048, -0.771)	94.6%
Erden *et al.* (2016)	76	10,506	9,907	-0.908	(-1.046, -0.77)	94.6%
Fischer *et al.*	76	10,480	9,881	-0.910	(-1.049, -0.771)	94.6%
Gezer *et al.*	76	10,535	9,936	-0.918	(-1.057, -0.78)	94.6%
Green *et al.*	76	10,492	9,898	-0.909	(-1.048, -0.771)	94.6%
Grilo *et al.*	76	10,420	9,821	-0.911	(-1.05, -0.772)	94.6%
Guedes *et al.*	76	10,507	9,919	-0.914	(-1.053, -0.775)	94.6%
Hancock *et al.*	76	10,526	9,927	-0.916	(-1.055, -0.777)	94.6%
Hayden *et al.*	76	10,299	9,700	-0.906	(-1.044, -0.768)	94.4%
Hosseini *et al.*	76	10,478	9,879	-0.911	(-1.05, -0.772)	94.6%
Ivezaj *et al.*	76	10,450	9,851	-0.910	(-1.049, -0.771)	94.6%
Järvholm *et al.*	76	10,494	9,906	-0.919	(-1.057, -0.78)	94.6%
Klemencic *et al.*	76	10,538	9,939	-0.921	(-1.059, -0.783)	94.6%
Leombruni *et al.*	76	10,519	9,920	-0.909	(-1.048, -0.771)	94.6%
Lier *et al.*	76	10,430	9,871	-0.920	(-1.058, -0.781)	94.6%
Malone *et al.*	76	10,448	9,902	-0.916	(-1.055, -0.777)	94.6%
Masheb *et al.*(2006)	76	10,412	9,813	-0.911	(-1.05, -0.772)	94.6%
Masheb *et al.*(2007)	76	10,420	9,821	-0.911	(-1.05, -0.772)	94.6%
Mathus-Vliegen *et al.*	76	10,508	9,909	-0.921	(-1.059, -0.783)	94.6%
Matini *et al.*	76	10,490	9,891	-0.932	(-1.065, -0.8)	94.0%
Musselman *et al.*	76	10,538	9,939	-0.914	(-1.053, -0.775)	94.6%
Ortega *et al.*	76	10,497	9,898	-0.923	(-1.061, -0.786)	94.5%
Papageorgiou *et al.*	76	10,504	9,923	-0.920	(-1.059, -0.782)	94.6%
Pasi *et al.* - RYGB	76	10,511	9,938	-0.923	(-1.06, -0.785)	94.5%
Pasi *et al.* - SG	76	10,511	9,932	-0.916	(-1.055, -0.777)	94.6%
Preiss *et al.*	76	10,458	9,870	-0.912	(-1.051, -0.773)	94.6%
Pyykko *et al.*	76	10,431	9,832	-0.925	(-1.062, -0.788)	94.4%
Ribeiro *et al.*	76	10,536	9,937	-0.912	(-1.051, -0.774)	94.6%
Rosenberger *et al.*	76	10,426	9,827	-0.909	(-1.048, -0.771)	94.6%
Sarwer *et al.*	76	10,525	9,927	-0.920	(-1.059, -0.782)	94.6%
Sellberg *et al.*	76	10,522	9,923	-0.909	(-1.048, -0.771)	94.6%
Strain *et al.* (2017)	76	10,519	9,936	-0.917	(-1.056, -0.779)	94.6%
Sysko *et al.*	76	10,456	9,857	-0.889	(-1.02, -0.759)	93.8%
Tan *et al.* - RYGB	76	10,537	9,938	-0.922	(-1.06, -0.784)	94.6%
Tan *et al.* - SG	76	10,522	9,923	-0.920	(-1.059, -0.782)	94.6%
Teufel *et al.*, Rieber *et al.* & Mack *et al.*	76	10,517	9,918	-0.915	(-1.054, -0.776)	94.6%
Thonney *et al.*	76	10,514	9,915	-0.895	(-1.029, -0.762)	94.2%
Tuli *et al.*	76	10,536	9,942	-0.920	(-1.058, -0.782)	94.6%

Uruc *et al.*	76	10,504	9,905	-0.907	(-1.045, -0.769)	94.6%
VanHout *et al.*	76	10,464	9,865	-0.920	(-1.059, -0.781)	94.6%
Vetrovsky *et al.*	76	10,531	9,932	-0.915	(-1.054, -0.776)	94.6%
Vreeken *et al.*	76	10,411	9,812	-0.915	(-1.054, -0.776)	94.6%
Wang *et al.*	76	10,527	9,928	-0.882	(-1.005, -0.758)	93.2%
Waters *et al.*	76	10,400	9,893	-0.921	(-1.059, -0.782)	94.6%
White *et al.* (2006)	76	10,418	9,819	-0.911	(-1.05, -0.772)	94.6%
White *et al.* (2010)	76	10,196	9,664	-0.920	(-1.059, -0.782)	94.4%
Winzer *et al.*	76	10,518	9,925	-0.920	(-1.058, -0.781)	94.6%
Zeller *et al.*	76	10,526	9,929	-0.913	(-1.052, -0.774)	94.6%
**Non-normally distributed studies**						
Removal of all studies where mean(3 SD) included negative values	9	405	398	-1.694	(-2.624, -0.764)	96.8%

Supplementary Table S5 Effect of sensitivity analysis on meta-analysis between baseline to long-term post-surgery (> 12 months)

Outcome of Interest	No. of studies	Total no. of patients		SMD	(95% CI)[b]	I^2%
		Pre-Surgery	*Post-Surgery*			
**Age**						
Removal of studies with only age ≤ 18 years	32	2,848	2,105	-0.697	(-0.86, -0.534)	85.5%
**Sex**						
Removal of female-only studies	32	2,826	2,081	-0.631	(-0.743, -0.520)	66.8%
**Quality Assessment**						
Good quality studies only	8	971	825	-0.599	(-0.812, -0.386)	76.5%
Good & fair quality studies only	27	2,393	1,854	-0.589	(-0.717, -0.461)	72.2%
**Questionnaire Used**						
BDI	20	1,397	1,066	-0.840	(-1.063, -0.617)	83.8%
CCEI	1	71	71	-0.130	(-0.458, 0.197)	-
HADS-D	7	883	755	-0.508	(-0.726, -0.289)	75.0%
HIS-GWB	1	157	18	-0.475	(-0.963, 0.013)	-
PHQ-9	2	250	120	-0.819	(-1.414, -0.224)	84.3%
SCL-90	3	184	174	-0.310	(-0.644, 0.024)	56.2%
YSR/ASR	1	19	12	-0.298	(-1.006, 0.410)	-
**One-sample removed analysis**						
Andersen *et al.* & Aasprang *et al.*	34	2,911	2,172	-0.669	(-0.823, -0.515)	83.8%
Atwood *et al.*	34	2,786	2,171	-0.665	(-0.818, -0.513)	83.4%
Blom-Hogestrol	34	2,798	2,053	-0.693	(-0.847, -0.538)	83.2%
Chalal-Kummen (2021)	34	2,712	2,009	-0.684	(-0.841, -0.527)	83.3%
Chalal-Kummen (2023)	34	2,733	2,007	-0.695	(-0.849, -0.542)	82.5%
Dixon *et al.* (2016)	34	2,812	2,090	-0.674	(-0.830, -0.518)	83.7%
Ferreira Pinto	34	2,901	2,156	-0.675	(-0.831, -0.520)	84.1%
Hafner	34	2,890	2,145	-0.697	(-0.849, -0.546)	83.1%
Hancock *et al.*	34	2,930	2,185	-0.678	(-0.833, -0.523)	84.3%
Järvholm *et al.*	34	2,898	2,131	-0.686	(-0.842, -0.530)	84.1%
Klemencic *et al.*	34	2,942	2,204	-0.688	(-0.842, -0.535)	84.1%
Kruseman	34	2,820	2,136	-0.695	(-0.848, -0.542)	83.2%
Mamplekou	34	2,902	2,158	-0.682	(-0.838, -0.526)	84.3%
Nandrino	34	2,825	2,080	-0.678	(-0.835, -0.522)	83.9%
Nickel	34	2,940	2,195	-0.679	(-0.833, -0.524)	84.3%
Pinto	34	2,901	2,156	-0.675	(-0.830, -0.519)	84.1%
Ryden	34	2,941	2,196	-0.682	(-0.836, -0.527)	84.3%
Sarwer *et al.*	34	2,929	2,185	-0.675	(-0.830, -0.520)	84.2%
Schowalter	34	2,833	2,176	-0.683	(-0.839, -0.527)	84.2%
Strain *et al.* (2014) - AGB	34	2,943	2,198	-0.676	(-0.831, -0.522)	84.3%
Strain *et al.* (2014) - BPD-DS	34	2,943	2,198	-0.670	(-0.823, -0.518)	84.0%
Strain *et al.* (2014) - RYGB	34	2,915	2,170	-0.671	(-0.826, -0.517)	84.0%
Strain *et al.* (2014) - SG	34	2,938	2,193	-0.681	(-0.836, -0.526)	84.3%
Strain *et al.* (2017)	34	2,923	2,194	-0.679	(-0.835, -0.524)	84.3%
Svanevik (GB)	34	2,907	2,172	-0.681	(-0.837, -0.525)	84.3%
Svanevik (SG)	34	2,906	2,169	-0.683	(-0.839, -0.528)	84.3%

Teufel, Rieber & Mack	34	2,894	2,119	-0.686	(-0.842, -0.530)	84.1%
Thonney *et al.*	34	2,918	2,173	-0.617	(-0.727, -0.507)	67.7%
Tuli *et al.*	34	2,940	2,195	-0.696	(-0.847, -0.545)	83.4%
VanHout *et al.*	34	2,868	2,123	-0.697	(-0.849, -0.545)	83.0%
Velcu	34	2,920	2,175	-0.654	(-0.799, -0.508)	81.9%
Waters *et al.*	34	2,804	2,198	-0.686	(-0.841, -0.531)	84.2%
White *et al.* (2010)	34	2,600	2,045	-0.686	(-0.843, -0.530)	83.2%
Wimmelmann	34	2,929	2,193	-0.695	(-0.847, -0.543)	83.5%
Zeller *et al.*	34	2,930	2,202	-0.681	(-0.836, -0.527)	84.3%
**Non-normally distributed studies**						
Removal of all studies where mean(3 SD) included negative values	2	2,802	2,057	-2.209	(-3.504, -0.914)	91.0%
